# Squalene Synthase As a Target for Chagas Disease Therapeutics

**DOI:** 10.1371/journal.ppat.1004114

**Published:** 2014-05-01

**Authors:** Na Shang, Qian Li, Tzu-Ping Ko, Hsiu-Chien Chan, Jikun Li, Yingying Zheng, Chun-Hsiang Huang, Feifei Ren, Chun-Chi Chen, Zhen Zhu, Melina Galizzi, Zhu-Hong Li, Carlos A. Rodrigues-Poveda, Dolores Gonzalez-Pacanowska, Phercyles Veiga-Santos, Tecia Maria Ulisses de Carvalho, Wanderley de Souza, Julio A. Urbina, Andrew H.-J. Wang, Roberto Docampo, Kai Li, Yi-Liang Liu, Eric Oldfield, Rey-Ting Guo

**Affiliations:** 1 Industrial Enzymes National Engineering Laboratory, Tianjin Institute of Industrial Biotechnology, Chinese Academy of Sciences, Tianjin, China; 2 Institute of Biological Chemistry, Academia Sinica, Taipei, Taiwan; 3 Center for Biophysics and Computational Biology, University of Illinois at Urbana-Champaign, Urbana, Illinois, United States of America; 4 Center for Tropical and Emerging Global Diseases and Department of Cellular Biology, University of Georgia, Athens, Georgia, United States of America; 5 Instituto de Parasitología y Biomedicina “Lopez-Neyra”, Consejo Superior de Investigaciones Cientificas, Granada, Spain; 6 Laboratório de Ultraestrutura Celular Hertha Meyer, CCS, Instituto de Biofísica Carlos Chagas Filho, Universidade Federal do Rio de Janeiro, Ilha do Fundão, Rio de Janeiro, Brazil; 7 Instituto Nacional de Ciência e Tecnologia em Biologia Estrutural e Bioimagens, Universidade Federal do Rio de Janeiro, Cidade Universitária, Ilha do Fundão, Rio de Janeiro, Brazil; 8 Diretoria de Programa, Instituto Nacional de Metrologia, Normalização e Qualidade Industrial–INMETRO, Duque de Caxias, Rio de Janeiro, Brazil; 9 Instituto Venezolano de Investigaciones Cientificas, Caracas, Venezuela; 10 Department of Chemistry, University of Illinois at Urbana-Champaign, Urbana, Illinois, United States of America; National Institute of Health, United States of America

## Abstract

Trypanosomatid parasites are the causative agents of many neglected tropical diseases and there is currently considerable interest in targeting endogenous sterol biosynthesis in these organisms as a route to the development of novel anti-infective drugs. Here, we report the first x-ray crystallographic structures of the enzyme squalene synthase (SQS) from a trypanosomatid parasite, *Trypanosoma cruzi*, the causative agent of Chagas disease. We obtained five structures of *T. cruzi* SQS and eight structures of human SQS with four classes of inhibitors: the substrate-analog S-*thiolo*-farnesyl diphosphate, the quinuclidines E5700 and ER119884, several lipophilic bisphosphonates, and the thiocyanate WC-9, with the structures of the two very potent quinuclidines suggesting strategies for selective inhibitor development. We also show that the lipophilic bisphosphonates have low nM activity against *T. cruzi* and inhibit endogenous sterol biosynthesis and that E5700 acts synergistically with the azole drug, posaconazole. The determination of the structures of trypanosomatid and human SQS enzymes with a diverse set of inhibitors active in cells provides insights into SQS inhibition, of interest in the context of the development of drugs against Chagas disease.

## Author Summary

Chagas disease is caused by the protozoan parasite *Trypanosoma cruzi* and affects eight million individuals, primarily in Latin America. Currently there is no cure for chronic *T. cruzi* infections. Unlike humans, this parasite use a variety of sterols (e.g. ergosterol, 24-ethyl-cholesta-5,7,22-trien-3 beta ol, and its 22-dihydro analogs), rather than cholesterol in their cell membranes, so inhibiting endogenous sterol biosynthesis is an important therapeutic target. Here, we report the first structure of the parasite's squalene synthase, which catalyzes the first committed step in sterol biosynthesis, as well as the structures of a broad range of squalene synthase inhibitors active against the clinically relevant intracellular stages, opening the way to new approaches to treating this neglected tropical disease.

## Introduction

**Figure 1:**
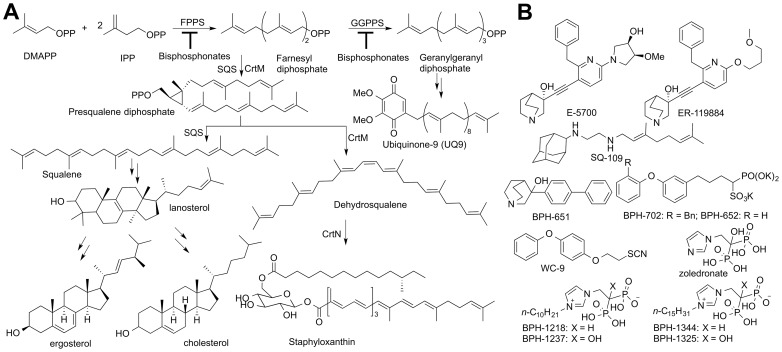
Biosynthetic pathways and structures of inhibitors. (A) Biosynthesis of ergosterol, cholesterol and staphyloxanthin. (B) Structures of inhibitors of interest.

Many millions of individuals are infected with the so-called “World's most neglected diseases”. These include the leishmaniases, with ∼12 million individuals affected [1], and in Latin America, Chagas disease. The latter affects ∼8 million individuals [2] including ∼300,000 in the United States, according to the US Centers for Disease Control and Prevention [3]). The global burden of Chagas disease is estimated to be ∼$7 billion a year [4]. There are no cures available for the chronic form of the disease which can involve cardiac myopathy, mega-oesophagus and mega-colon, although clinical trials with the azole drug posaconazole and a ravuconazole prodrug are in progress [5], [6]. Both of these compounds function by blocking the ergosterol biosynthesis pathway [7], [8] shown in Figure 1A, as described in a recent review [9].

Ergosterol is an essential membrane sterol in many trypanosomatid parasites and plays the same structural role as does cholesterol in humans. It is synthesized in a lengthy series of reactions beginning with the condensation of dimethylallyl diphosphate (DMAPP, Figure 1A) with two molecules of iso-pentenyl diphosphate (IPP) to form farnesyl diphosphate (FPP) in a reaction catalyzed by farnesyl diphosphate synthase (FPPS), a reaction that is inhibited by bisphosphonate drugs [10]. Two FPP molecules then condense in a “head-to-head” fashion to form presqualene diphosphate (the “first-half” reaction) which then undergoes loss of diphosphate, rearrangement, and reduction by NADPH to form squalene (the “second-half” reaction), Figure 1A [11], both reactions being catalyzed by squalene synthase (SQS). Squalene is epoxidized (by O_2_/squalene epoxidase) to form oxidosqualene, which is then electro-cyclized by oxidosqualene cyclase (OSC) to form lanosterol. Lanosterol is demethylated by the 14-α demethylase/P450 system (CYP51), the target of the azole drugs, and after several more steps, ergosterol, 24-ethyl-cholesta-5,7,22-trien-3-β-ol, and its 22-dihydro analogs are produced. Yeasts and fungi also produce ergosterol and the azole drugs were originally developed as anti-fungals [12] but were later found to have potent activity against *T. cruzi* [7], [13]. More recently, SQS inhibitors, quinuclidines (Figure 1B), originally developed as cholesterol-lowering drug leads [14], have also been found to kill *T. cruzi*, *in vitro* and *in vivo* [15]. However, more selective SQS inhibitors are of interest since they would reduce potential side-effects on steroidogenesis [16]. To begin to contemplate how to design such selective quinuclidine species, it is desirable to first learn more about how these compounds inhibit both human and trypanosomatid SQS, but to date, no such structures have been reported.

There is also interest in the development of SQS inhibitors with completely different structures and properties, including compounds that might have multiple sites of action in the ergosterol biosynthesis pathway (polypharmacology), as well as different tissue distributions. Other SQS inhibitors that have been discovered include the thiocyanate WC-9 [17] as well as the bisphosphonates ibandronate and incadronate. These bisphosphonates inhibit both human SQS (HsSQS) and HsFPPS [18]–[20] and block cholesterol biosynthesis [19]. Ibandronate is used clinically to treat osteoporosis and functions by inhibiting FPP biosynthesis in osteoclasts. Unfortunately, ibandronate binds tightly to human bone mineral [21] so this so-called nitrogen-containing bisphosphonate would not be a good anti-infective lead since it is rapidly removed from the circulation, but more lipophilic bisphosphonates [22]–[24] have poorer bone-binding capacity and have been shown to kill parasitic protozoa, such as malaria parasites (*Plasmodium spp.*), both *in vitro* and *in vivo* [23], [24]. In malaria parasites, unlike the situation with *T. cruzi*, there is no squalene synthase, and cell growth inhibition by lipophilic bisphosphonates is primarily at the level of FPPS/GGPPS (geranylgeranyl diphosphate synthase) inhibition [23].

The structure of human SQS has been reported [25] but gave little insight into the SQS mechanism of action. More recently, we reported [26] the structures of a bacterial SQS homolog, dehydrosqualene synthase (CrtM) from *Staphylococcus aureus* which carries out the same first-half reaction as does SQS, formation of presqualene diphosphate (PSPP, Figure 1A) from FPP. With CrtM, PSPP then loses diphosphate, and the resulting carbocation rearranges and loses a proton to form dehydrosqualene, and we obtained a quinuclidine inhibitor-bound structure, proposed to mimic one of the carbocation intermediates in catalysis [27]. Based on these results and those of others [28], [29] the SQS mechanism of action shown in Figure S1 is suggested. There have, however, been no structures of any trypanosomatid SQS enzyme. Here, we report the structures of human SQS and *T. cruzi* SQS bound to a substrate-like inhibitor (S-*thiolo*-farnesyldiphosphate, FSPP), as well as the structures of both enzymes bound to two potent quinuclidine inhibitors (E5700 and ER119884, Figure 1B) which suggest routes to selective inhibitor development. We also report six x-ray structures of lipophilic bisphosphonate inhibitors bound to TcSQS and/or HsSQS, as well as the activity of a series of lipophilic bisphosphonates against *T. cruzi* FPPS, TcSQS, and solanesyl diphosphate synthase (TcSPPS, involved in ubiquinone-9 biosynthesis, Figure 1A), and against *T. cruzi* amastigotes, plus, we demonstrate synergistic effects of E5700 and posaconazole against amastigotes.

## Results and Discussion

### Structures of *T. cruzi* and human squalene synthase bound to FSPP

We expressed, purified and crystallized *T. cruzi* squalene synthase and solved its structure using the method of molecular replacement. TcSQS crystals could only be obtained in the presence of the substrate-like inhibitor, FSPP. Full experimental details are given in Materials and Methods. The construct was the recombinant enzyme described previously [16] in which 24 amino acids from the membrane-spanning N-terminus and 36 from the C-terminus were deleted in order to enable high-level production of active, soluble protein. We also crystallized HsSQS [30] and solved its structure, again in the presence of FSPP. Full crystallographic data acquisition and refinement statistics for both proteins are given in Tables 1 and 2. For the 13 structures investigated the resolution was in the range 2.06–3.00 Å (2.48 Å, on average), Tables 1–3.

**Table 1:** Summary of data processing and refinement statistics of TcSQS complex crystals.

Name	TcSQS-FSPP	TcSQS-E5700	TcSQS-ER119884	TcSQS-BPH1237	TcSQS-BPH1344
PDB code	3WCA	3WCC	3WCE	3WCB	3WCG
***Data collection***					
Resolution (Å)	25-2.24 (2.32-2.24)	25-2.32 (2.40-2.32)	25-2.75 (2.85-2.75)	25-3.00 (3.11-3.00)	25-2.80 (2.90-2.80)
Space group	*P*2_1_2_1_2_1_	*P*2_1_2_1_2_1_	*P*2_1_2_1_2_1_	*P*2_1_2_1_2_1_	*P*2_1_2_1_2_1_
Unit-cell					
*a*/*b*/*c* (Å)	79.13/132.87/141.92	79.07/131.60/143.12	78.81/131.42/142.23	79.40/127.69/142.56	83.09/128.97/144.97
No. of reflections Measured	541693 (53169)	339484 (33701)	184672 (18170)	127795 (11833)	171915 (15479)
Unique	72859 (7185)	65344 (6481)	39259 (3866)	29564 (2752)	39223 (3518)
Completeness (%)	100.0 (100.0)	99.6 (100.0)	100.0 (100.0)	99.1 (94.6)	98.6 (90.1)
*R*_merge_ (%)^a^	5.4 (46.2)	6.1 (48.8)	6.8 (47.9)	7.6 (48.9)	5.6 (41.0)
Mean I/σ(I)	44.6 (5.4)	34.1 (4.2)	25.4 (4.0)	15.1 (1.6)	23.7 (2.1)
Multiplicity	7.4 (7.5)	5.2 (5.2)	4.7 (4.8)	4.3 (4.1)	4.4 (3.9)
Wilson B	44.4	44.5	55.8	50.0	60.3
***Refinement***					
*R_work_* (%)	21.5 (26.3)	21.8 (28.0)	22.7 (29.6)	22.7 (34.1)	23.6 (38.9)
*R_free_* (%)	26.2 (31.1)	25.7 (31.7)	27.6 (34.7)	23.7 (33.3)	26.5 (37.4)
Geometry deviations					
Bond lengths (Å)	0.006	0.006	0.003	0.003	0.004
Bond angles (°)	1.10	1.17	0.89	0.91	0.94
Ramachandran plot (%)					
Most favored	95.0	95.0	92.8	94.6	93.5
allowed	4.6	4.3	6.6	4.9	6.1
Disallowed	0.4	0.7	0.6	0.5	0.4
Mean B-values(Å^2^)/No. of atoms					
Protein	53.6/11005	52.4/11013	62.6/11001	74.4/11013	78.0/11013
Solvent	54.4/741	52.1/502	49.7/350	51.9/196	55.4/173
Ligand	79.9/123	43.1/128	91.2/120	121.7/64	112.6/120

Values in parentheses are for the highest resolution shell.

a *R*_merge_ = ∑*_hkl_*∑_i_|*I*_i_(*hkl*)−<*I*(*hkl*)>|/∑*_hkl_*∑_i_ *I*_i_(*hkl*).

**Table 2:** Summary of data processing and refinement statistics of HsSQS complex crystals.

Name	HsSQS-FSPP	HsSQS-E5700	HsSQS-ER119884	HsSQS-WC-9
PDB code	3WC9	3WCJ	3WCM	3WCD
***Data collection***				
Resolution (Å)	25-2.82 (2.92-2.82)	25-2.20 (2.28-2.20)	25-2.06 (2.13-2.06)	25-2.75 (2.85-2.75)
Space group	*P*2_1_	*P*2_1_	*P*2_1_	*P*2_1_
Unit-cell				
*a*/*b*/*c* (Å)	85.19/153.33/90.99	85.33/153.63/92.12	85.62/153.45/92.12	85.73/153.74/91.43
*β* (°)	91.84	90.86	90.86	91.00
No. of reflections Measured	221507 (22296)	387940 (38371)	855441 (84900)	233294 (23130)
Unique	55674 (5574)	120906 (11991)	146577 (14638)	61519 (6087)
Completeness (%)	99.7 (99.4)	99.8 (99.9)	100.0 (100.0)	100.0 (100.0)
*R*_merge_ (%)^a^	11.4 (48.3)	8.0 (46.0)	8.2 (49.3)	9.3 (45.8)
Mean I/σ(I)	17.9 (4.6)	19.3 (3.1)	27.3 (4.2)	18.5 (3.7)
Multiplicity	4.0 (4.0)	3.2 (3.2)	5.8 (5.7)	3.8 (3.8)
Wilson B	24.2	31.7	26.6	35.7
Refinement				
*R_work_* (%)	18.4 (26.7)	19.8 (28.4)	18.6 (25.1)	19.6 (28.9)
*R_free_* (%)	24.8 (32.0)	22.5 (30.9)	22.2 (28.0)	24.9 (35.5)
Geometry deviations				
Bond lengths (Å)	0.007	0.007	0.009	0.004
Bond angles (°)	1.20	1.14	1.19	0.90
Ramachandran plot (%)				
Most favored	93.6	95.9	96.9	93.2
allowed	5.7	3.6	3.0	6.0
Disallowed	0.7	0.5	0.1	0.8
Mean B-values (Å^2^)/No. of atoms				
Protein	42.9/16168	43.8/16183	37.5/15965	49.1/16177
Solvent	34.6/833	43.4/825	40.7/1136	44.0/768
Ligand	69.7/168	39.9/192	38.0/180	92.1/76

Values in parentheses are for the highest resolution shell.

a *R*_merge_ = ∑*_hkl_*∑_i_|*I*_i_(*hkl*)−<*I*(*hkl*)>|/∑*_hkl_*∑_i_ *I*_i_(*hkl*).

**Table 3:** Summary of data processing and refinement statistics of HsSQS complex crystals.

Name	HsSQS-BPH1218	HsSQS-BPH1237	HsSQS-BPH1325	HsSQS-BPH1344
PDB code	3WCF	3WCH	3WCI	3WCL
***Data collection***				
Resolution (Å)	25-2.22 (2.30-2.22)	25-2.50 (2.59-2.50)	25-2.30 (2.38-2.30)	25-2.24 (2.32-2.24)
Space group	*P*2_1_	*P2*_1_	*P2*_1_	*P2*_1_
Unit-cell				
*a*/*b*/*c* (Å)	85.18/153.19/90.11	85.63/153.55/90.40	85.38/153.62/91.16	85.25/153.30/90.93
*β* (°)	92.37	91.18	90.82	91.48
No. of reflections Measured	679240 (63361)	308267 (30647)	380278 (37204)	671951 (64477)
Unique	111130 (10387)	81157 (8065)	102588 (10055)	110868 (10570)
Completeness (%)	98.3 (92.0)	99.8 (99.1)	99.3 (97.6)	99.2 (94.7)
*R*_merge_ (%)^a^	5.8 (27.1)	9.1 (42.7)	7.9 (44.0)	7.4 (32.7)
Mean I/σ(I)	30.1 (3.6)	17.0 (4.4)	27.3 (4.7)	28.0 (3.7)
Multiplicity	6.1 (5.2)	3.8 (3.9)	3.7 (3.6)	6.1 (5.2)
Wilson B	37.1	37.6	34.2	34.2
Refinement				
*R_work_* (%)	22.4 (29.9)	19.2 (25.1)	21.7 (28.3)	18.8 (27.9)
*R_free_* (%)	20.4 (28.0)	25.0 (32.3)	25.3 (33.2)	22.9 (32.4)
Geometry deviations				
Bond lengths (Å)	0.020	0.020	0.006	0.006
Bond angles (°)	1.82	1.86	1.15	1.09
Ramachandran plot (%)				
Most favored	96.1	95.4	95.7	95.5
allowed	3.4	3.9	3.9	4.0
Disallowed	0.5	0.7	0.4	0.6
Mean B-values (Å^2^)/No. of atoms				
Protein	48.8/16177	47.7/16177	43.9/16177	43.4/16177
Solvent	41.0/495	42.1/427	48.8/1370	43.1/1374
Ligand	74.6/150	79.6/217	99.0/186	98.7/30

Values in parentheses are for the highest resolution shell.

a *R*_merge_ = ∑*_hkl_*∑_i_|*I*_i_(*hkl*)−<*I*(*hkl*)>|/∑*_hkl_*∑_i_ *I*_i_(*hkl*).

**Figure 2:**
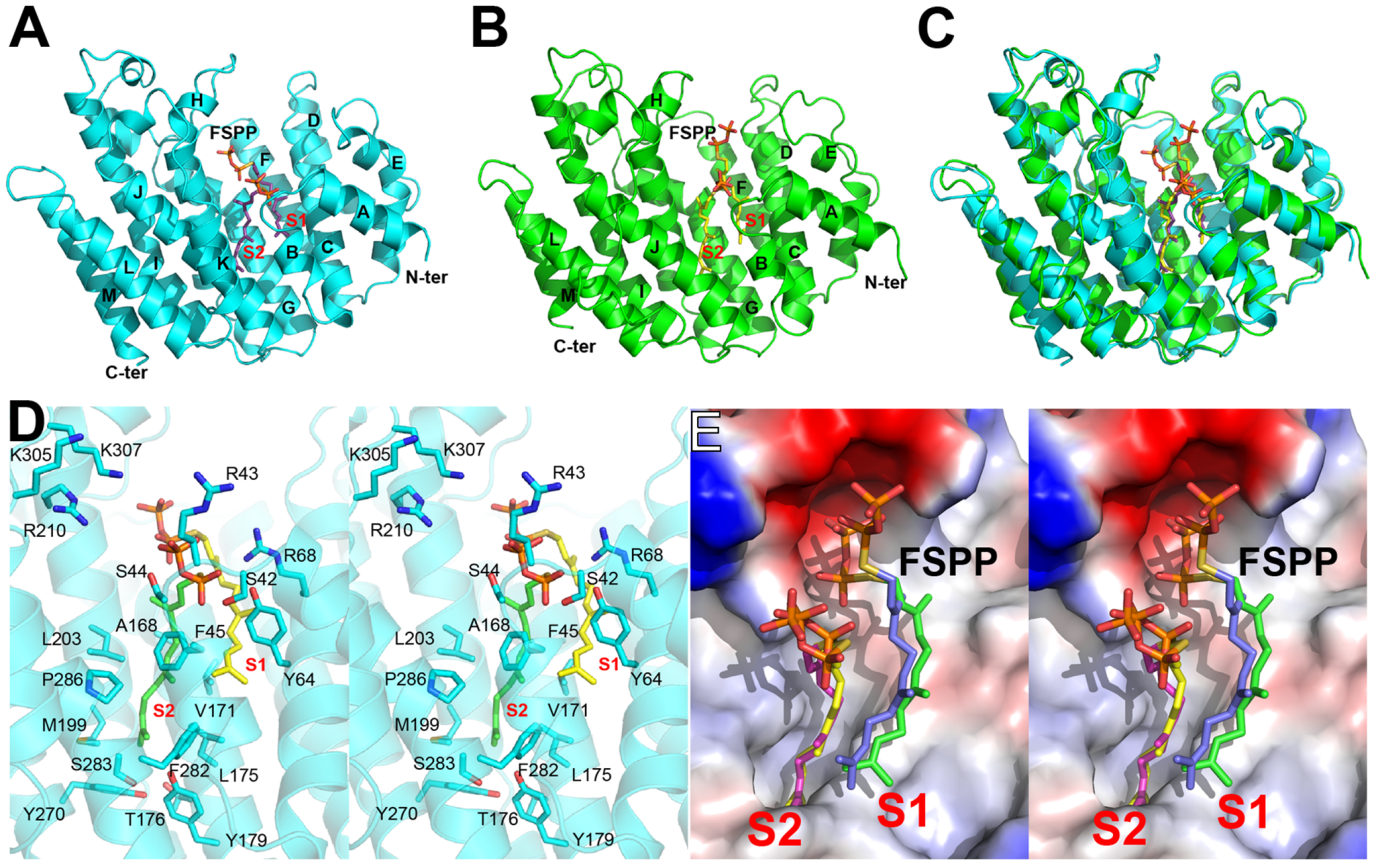
Structural comparisons between TcSQS and HsSQS. (A) Overall structure of TcSQS in complex with FSPP. TcSQS and FSPP are shown in cyan ribbon and purple sticks, respectively. (B) As (A) but for HsSQS in green ribbon (SQS) and yellow sticks (FSPP). (C) Superimposition of TcSQS/FSPP (cyan/purple) and HsSQS/FSPP (green/yellow) structures. (D) Stereo view of FSPP and TcSQS amino-acid residues. FSPP ligands are in purple sticks. (E) Superimposition of FSPP ligands in TcSQS (green and yellow) and HsSQS (blue and magenta).

All TcSQS crystals investigated belong to the space group P2_1_2_1_2_1_ and contain four molecules in the asymmetric unit. The crystals of HsSQS belong to the space group P2_1_ and contain six HsSQS molecules in the asymmetric unit. The two enzymes share 44% amino acid identity and have the same fold (Figure 2A, B). The root-mean-square deviation (RMSD) is 0.87 Å for 298 matched Cα atoms within 2 Å of each other. A superposition of the TcSQS and HsSQS structures is shown in Figure 2C. Two FSPP substrate-analog inhibitor molecules bind to one TcSQS monomer. One FSPP is located in the allylic diphosphate (prenyl acceptor) site S1 previously observed in CrtM, Figure 2D, and the FPP that is expected to bind to this site is involved in the first diphosphate ionization reaction. Electron densities are in Figure S2A and (expanded) in Figure S3A. The other is located in the homo-allylic (prenyl donor) site, S2. The hydrocarbon moiety in the FSPP in S2 interacts with Ala168, Val171, Gly172, Leu175, Thr176 and Tyr179 from helix αG; Met199, Gly200 and Leu203 from helix αH; Phe282, Ser283 and Pro286 from helix αJ, as well as Phe45, Tyr64 and Tyr270. The diphosphate group of FSPP in S2 is located at the N-terminal end of helix αB and interacts with Ser42, Arg43, Ser44, and Arg68, while the diphosphate group from the first FSPP (in S1) is adjacent to the DXXED motif in helix αH. The DXXXD motif is highly conserved in many terpene synthases and cyclases [31] and is involved in binding to, in general, 3 Mg^2+^ that in turn bind to an allylic diphosphate, the Mg^2+^ facilitating carbocation formation due to diphosphate loss [32]. In HsSQS, the hydrocarbon moieties of FSPP bind in a similar way to the S1 and S2 sites, Figure 2E, but the orientations of the diphosphate head groups are somewhat different to those found with TcSQS, as can be seen in the ligand superpositions shown in Figure 2E, although the IC_50_ values for SQS inhibition are quite similar: 31 nM (TcSQS) and 64 nM (HsSQS). Binding appears to be driven by primarily hydrophobic interactions, consistent with limited electrostatic interactions with cationic amino-acid residues and the relatively solvent-accessible positions of the diphosphate group. Isothermal titration calorimetry results for the binding of FSPP to TcSQS are shown in Figure S4 and indicate a *K*_d_ of 2.6 µM. All major protein-ligand interactions are shown in Figure S5A–D.

### Structures of SQS with quinuclidine inhibitors

**Figure 3:**
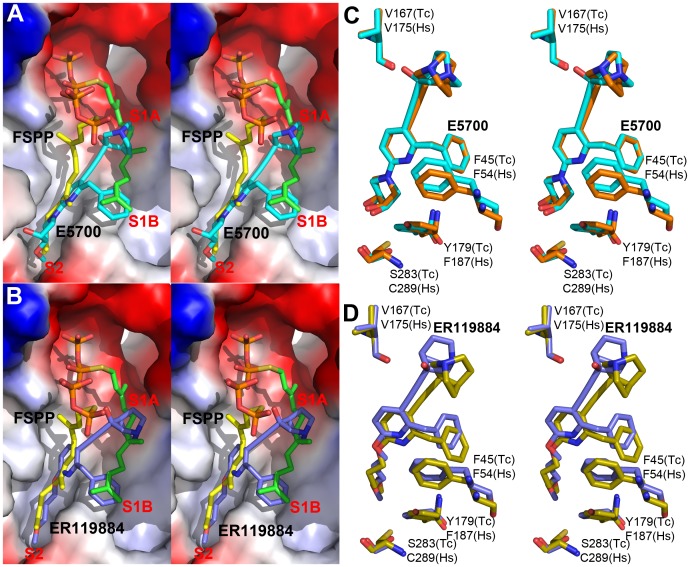
Crystal structures of E5700 and ER119884 bound to TcSQS and HsSQS. (A) TcSQS/E5700 (PDB ID code 3WCC) superimposed on TcSQS/FSPP (PDB ID code 3WCA), E5700 in cyan, FSPP in green and yellow. (B) TcSQS/ER119884 (PDB ID code 3WCE) superimposed on TcSQS/FSPP (PDB code ID 3WCA). ER119884 in blue, FSPP in green and yellow. (C) Superimposed Tc/Hs SQS structures with E5700 (PDB ID codes 3WCC and 3WCJ), showing protein residues of interest. TcSQS in cyan, HsSQS in orange. (D) Superimposed Tc/Hs SQS structures with ER119884 (PDB ID codes 3WCE and 3WCM), showing protein residues of interest. TcSQS in blue, HsSQS in yellow.

We next obtained the structures of two very potent SQS inhibitors: the quinuclidines E5700 and ER119884 (Figure 1B) developed by Eisai Company (Tokyo, Japan) as anti-hypercholesterolemia drug leads that also have ∼1 nM activity against TcSQS and potent antiproliferative activity against *T. cruzi* [15], [16]. We solved the structures of the quinuclidines bound to both TcSQS and HsSQS using the method of molecular replacement. Full data acquisition and structure refinement details are in Tables 1 and 2 and electron densities are in Figures S2, S6 and S7. In both proteins the inhibitors span both the S1 and S2 isoprenoid binding sites, helping explain the very potent enzyme inhibition seen experimentally. With E5700, the quinuclidine ring occupies the S1 carbocation site (called here S1A) while the opposite end of the inhibitor is located at the bottom end of the S2 binding site, Figure 3A. The quinuclidine is expected to bind as a cationic species (quinuclidine pK_a_ ∼10), and likely mimics one of the carbocations involved in squalene biosynthesis. The phenyl ring in the benzyl group occupies the bottom of the S1 binding site (S1B), as shown in Figure 3A, and the terminal methoxyl group in E5700 (in S2) is closely aligned with the “tail” of the FSPP ligand (in S2). With the ER119884 inhibitor bound to TcSQS (Figure 3B), the quinuclidine again binds to S1A and, as with E5700, the benzyl group occupies S1B, at the terminus of the S1 FPP site. The methyl terminus of the methoxy-propyl group also again aligns with the “tail” of the FSPP in S2 (Figure 3B). Detailed protein-ligand interactions are in Figure S5E–H.

With the HsSQS/quinuclidine structures we were able to obtain crystals by co-crystallization. However, this was not possible with TcSQS and it was necessary to soak TcSQS⋅FSPP crystals with the quinuclidine inhibitors, raising the possibility that there might be incomplete FSPP replacement (even in the presence of 10 mM quinuclidine). We thus show in Figures S3, S5 and S7 expanded and stereo views of the ligands in the TcSQS structures (FSPP, E5700 and ER119884) and for comparison, the HsSQS/quinuclidine densities (Figure S6), all contoured at 3σ. As can be seen in the TcSQS structures (superimposed on the TcSQS⋅FSPP structure), the ligand densities are well defined for all of E5700 (Figure S7A) and for almost all of ER119884 (Figure 3C and S7B).

To begin to see how selective inhibitors might be designed, we obtained the structures of both E5700 and ER119884 bound to HsSQS. Data acquisition and structure refinement details are given in Table 2. We show in Figure 3C a superposition of E5700 bound to TcSQS (cyan) with that of E5700 bound to human SQS (orange). It is clear that the drug binds to the two enzymes in a generally similar way, albeit with a slight tilt of the long axis. The quinuclidinol cage is also rotated by ∼70° between the two structures, changing the OH position by 2.4 Å and the cation center by 1.4 Å. More intriguing is the observation that at the bottom of the S2 ligand-binding pocket, TcSQS has Tyr179/Ser283 while HsSQS has Phe187/Cys289. In the future it may be possible to design an inhibitor that will not bind to Phe187/Cys289 in HsSQS, but will H-bond to Tyr179/Ser283 in TcSQS, since Tyr/Ser are good H-bond acceptors while Cys (and of course Phe) are not [33], [34]. The second quinuclidine, ER119884, binds in basically the same way as does E5700, with the quinuclidinol and benzyl groups in S1 while the methoxypropyl side chain locates to S2, as shown in Figure 3D. And as with the E5700 structures, the ER119884 structures indicate close proximity to the F/Y and C/S residues that may be targets for more selective inhibitors.

### Lipophilic bisphosphonates are potent SQS inhibitors

We next sought to find new SQS inhibitors since, although having extremely potent activity against TcSQS and *T. cruzi*, the quinuclidines have not yet provided parasitological cures *in vivo*, due most likely to a short mean residence time [15], so new leads are of interest. In earlier work, Amin et al. [18] reported that human SQS was inhibited by nitrogen-containing bisphosphonate drugs such as ibandronate and incadronate, with low nM *K*_i_ values. Such bisphosphonates are also potent FPPS inhibitors but are extremely polar (clog*P*∼−3) and do not readily enter cells with Amin et al. reporting only a ∼60 µM IC_50_ in cholesterol lowering in J774 cells [19]. In addition, as noted previously, such bisphosphonates bind avidly to bone mineral – a desirable feature for a bone resorption drug but not, in general, an anti-infective. The binding of bisphosphonates to bone involves the interaction of the phosphonate groups, the 1-OH group, as well as of an unhindered cationic side-chain with the bone surface [21]. Replacement of the 1-OH group by a 1-H reduces bone binding, as does introduction of a hydrophobic side-chain which also greatly increases activity in a variety of cell assays including γδ T cell activation (where FPPS targeting is involved) [35], tumor cell killing [22] and inhibition of malaria parasite cell growth [24].

**Figure 4:**
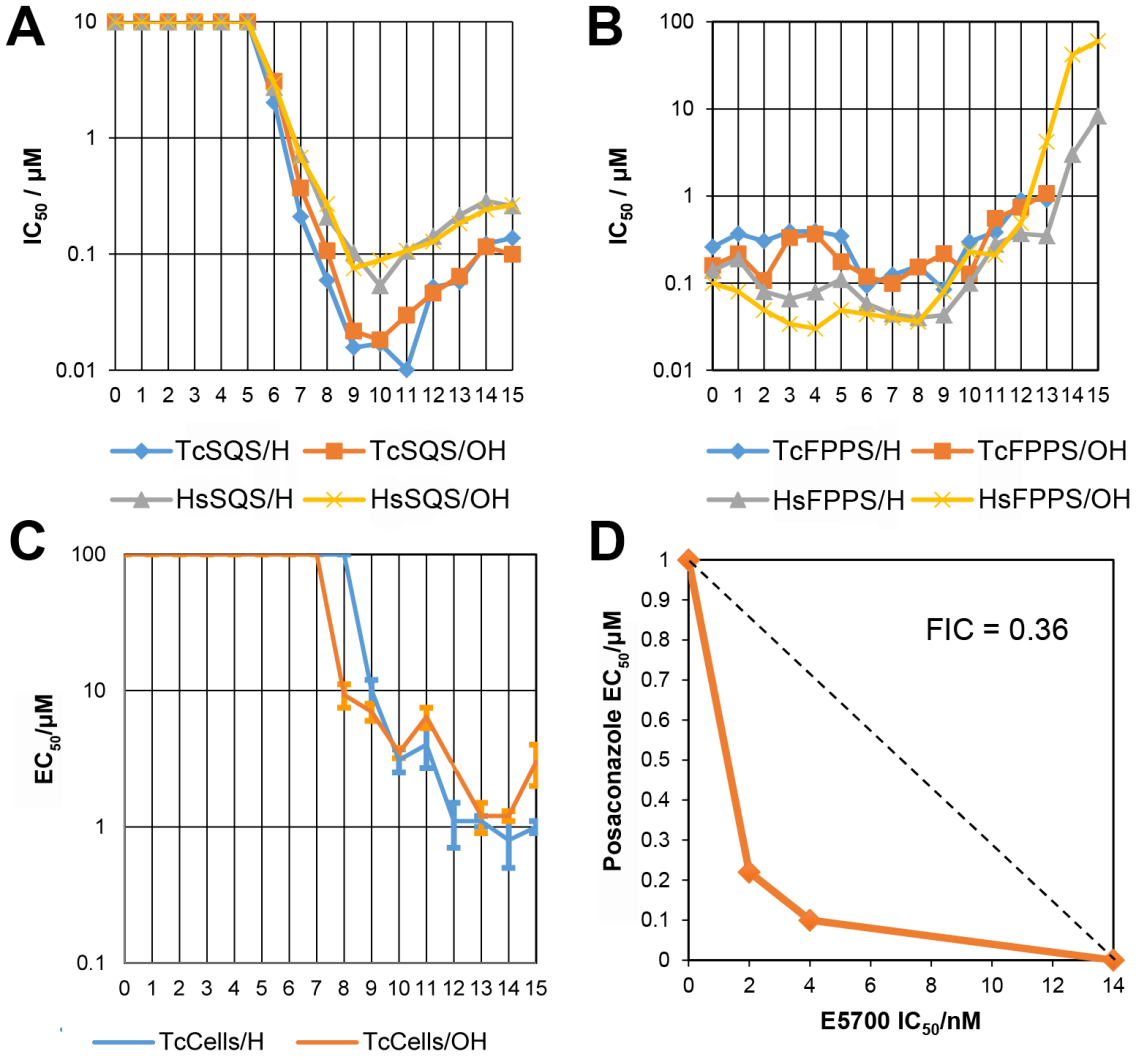
Enzyme and cell growth inhibition results. (A) TcSQS and HsSQS inhibition by bisphosphonates as a function of n, the number of carbons in the n-alkyl group. For n<6, the IC_50_ values are larger than 10 µM; (B) As (A) but for TcFPPS and HsFPPS. (C) *T. cruzi* amastigote cell growth inhibition by bisphosphonates as a function of n. (D) Isobologram showing the synergistic effect of E5700 and posaconazole against *T. cruzi* amastigotes after 96 h of treatment. Dashed line corresponds to the predicted positions of the experimental points for additive effects.

We thus next tested a series of lipophilic bisphosphonates [24], n-alkyl side-chain analogs of zoledronate (Figure 1B), with or without 1-OH groups (part of the “bone-hook” that enables binding to bone mineral) against TcSQS, HsSQS and TcFPPS (HsFPPS inhibition being reported earlier [24]). We also tested a subset of these bisphosphonates for their ability to inhibit the proliferation of the clinically relevant, intracellular, amastigote form of *T. cruzi*. Results are shown in Table 4 and Figure 4A. As can be seen in Figure 4A, we find with SQS inhibition that there is an increase in potency with increasing chain length up to n = 10–11, but then potency decreases on further chain elongation, for both TcSQS and HsSQS. There is no obvious effect of the 1-OH/1-H group on enzyme inhibition, but for the most active (C_11_) species, the HsSQS IC_50_ values are ∼10× higher than with TcSQS, indicating selectivity for the parasite enzyme. With TcFPPS, the increase in activity with chain length is less pronounced (as found also with HsFPPS), and maximum activity is found with a C_6_ substituent while the longer chain species are notably less active, Figure 4B. Activity in *T. cruzi* cell growth inhibition increases monotonically with increasing chain length up to the longest (C_15_) chain species investigated, Figure 4C, as seen also with malaria parasite cell growth inhibition [24], an effect that we attribute [36] in both organisms to the improved log*P* values (clog*P*∼3.3–3.8 for the longer chain inhibitors), resulting in enhanced cell permeability. FPP biosynthesis is not likely to be a major target with the most effective cell growth inhibitors (the C_10_–C_15_ bisphosphonates) in *T. cruzi* since they have relatively weak activity against FPPS and there was no “rescue” afforded by farnesol, and only a ∼20% rescue by geranylgeraniol (at 40 µM) with BPH-1218, one of the most potent SQS inhibitors. However, in the presence of BPH-1218 (C_10_, 4 µM), the content of exogeneously-acquired cholesterol in *T. cruzi* epimastigotes increased from 51% to 71% of total sterols, while that of endogenous ergosterol and the three major ergosterol related sterols (24-ethyl-5,7,22-cholestatrien-3β-ol, ergosta-5,7-diene-3β-ol and 24-ethyl-5,7- cholestadien-3β-ol) decreased from 36% to 15% of total sterols, Figure S8A. The lipophilic bisphosphonates are thus potent SQS inhibitors that target sterol biosynthesis and are active in cells. The longer chain compounds are also poorer inhibitors of TcFPPS (Figure 4B), HsFPPS, a *Plasmodium* FPPS/GGPPS [24] as well as HsGGPPS (IC_50_ = 2 µM for the C_12_/1-H species [23], although any ability to inhibit TcFPPS in addition to TcSQS is of potential interest since this could result in enhanced efficacy in growth inhibition because FPPS inhibition would yield less FPP for the SQS-catalyzed reaction. Importantly, the most potent *T. cruzi* cell growth inhibitors had low cytotoxicity against Vero cells, Table 4.

**Table 4:** IC_50_ values (in µM) for lipophilic bisphosphonates inhibiting *T. cruzi* and human SQS and FPPS, *T. cruzi* amastigote cell growth and Vero (host) cell growth^a^.

BPH #	Structure	TcSQS	HsSQS	TcFPPS	HsFPPS	*T. cruzi* amastigotes^b^^,^ c	Vero cells^d^
91	C-0,OH	>10	>10	0.16	0.10	>10	N.D.
1223	C-1,OH	>10	>10	0.22	0.080	>10	N.D.
1335	C-2,OH	>10	>10	0.11	0.049	>10	N.D.
1336	C-3,OH	>10	>10	0.33	0.034	>10	N.D.
1260	C-4,OH	>10	>10	0.37	0.030	>10	N.D.
1324	C-5,OH	>10	>10	0.18	0.049	>10	N.D.
1239	C-6,OH	3.1	3.0	0.12	0.044	>10	N.D.
1323	C-7,OH	0.37	0.68	0.09	0.040	>10	N.D.
1222	C-8,OH	0.11	0.27	0.15	0.036	9.3±1.8	50 ±1.4
1238	C-9,OH	0.021	0.075	0.22	0.080	7.0±1.0	190±40
1237	C-10,OH	0.018	0.089	0.13	0.23	3.5±0.3	380±100
1236	C-11,OH	0.030	0.11	0.55	0.21	6.4±1.1	460±250
1269	C-12,OH	0.046	0.13	0.74	0.49	3.0±0.02	>125
1322	C-13,OH	0.064	0.18	1.07	4.2	1.2±0.3	100±40
1338	C-14,OH	0.12	0.24	>5	42	1.2±0.1	51±10
1325	C-15,OH	0.10	0.27	>5	60	3.0±1.0	65±18
301	C-0,H	>10	>10	0.26	0.14	>10	N.D.
1224	C-1,H	>10	>10	0.37	0.19	>10	N.D.
1227	C-2,H	>10	>10	0.31	0.08	>10	N.D.
1337	C-3,H	>10	>10	0.39	0.066	>10	N.D.
1215	C-4,H	>10	>10	0.39	0.079	>10	N.D.
1339	C-5,H	>10	>10	0.35	0.11	>10	N.D.
1216	C-6,H	2.0	2.7	0.09	0.058	>10	N.D.
1326	C-7,H	0.21	0.72	0.12	0.044	>10	N.D.
1217	C-8,H	0.06	0.21	0.16	0.040	>10	N.D.
1327	C-9,H	0.016	0.10	0.085	0.043	10±2	∼1 mM
1218	C-10,H	0.017	0.053	0.30	0.10	3.1±0.6	∼1 mM
1328	C-11,H	0.010	0.11	0.39	0.28	4.0±1.3	∼1 mM
703	C-12,H	0.052	0.14	0.90	0.37	1.1±0.4	∼1 mM
1329	C-13,H	0.058	0.22	0.91	0.35	1.1±0.1	∼1 mM
1219	C-14,H	0.12	0.29	>5	3.0	0.8±0.3	∼1 mM
1344	C-15,H	0.14	0.26	>5	8.3	1.0±0.1	∼1 mM

a N. D. = not determined.

b Results are means ± SD from 2 or 3 independent experiments.

c Benznidazole IC_50_ = 1.9±0.9 µM.

d Benznidazole IC_50_∼1 mM.

There could of course be other targets for the lipophilic bisphosphonates, the most likely being solanesyl diphosphate synthase (SPPS) which produces the C_45_ diphosphate used in UQ_9_ (ubiquinone-9) biosynthesis [37]. We tested a subset of inhibitors against SPPS finding IC_50_s as low as 60 nM for the C_10_ species. However, unlike the situation with SQS, which is involved in production of the essential ergosterol not produced by humans, it is possible that SPPS is not an “essential gene” for *T. cruzi* in amastigotes, since quinones might be obtained from the host cell. There is, however, a ∼40% decrease in UQ_9_ biosynthesis with BPH-1218 in epimastigotes, consistent with multi-site targeting.

We thus believe that reduction in ergosterol (and related endogenous sterols) is a major contributor to the inhibition of cell growth. How sterol composition affects cell growth at the molecular level is not known. However, the azole class [38], [39] of drugs that target ergosterol biosynthesis in fungi have been shown to lead to progressive alkalinization of yeast vacuoles and a loss of V-ATPase activity. While not yet examined in trypanosomatids, this may also be the ultimate mode of action of this class of inhibitors, and since the quinuclidines target ergosterol biosynthesis [40], disruption of V-ATPase function, as found with the azoles in fungi, may also be important.

### Structures of lipophilic bisphosphonates bound to SQS

**Figure 5:**
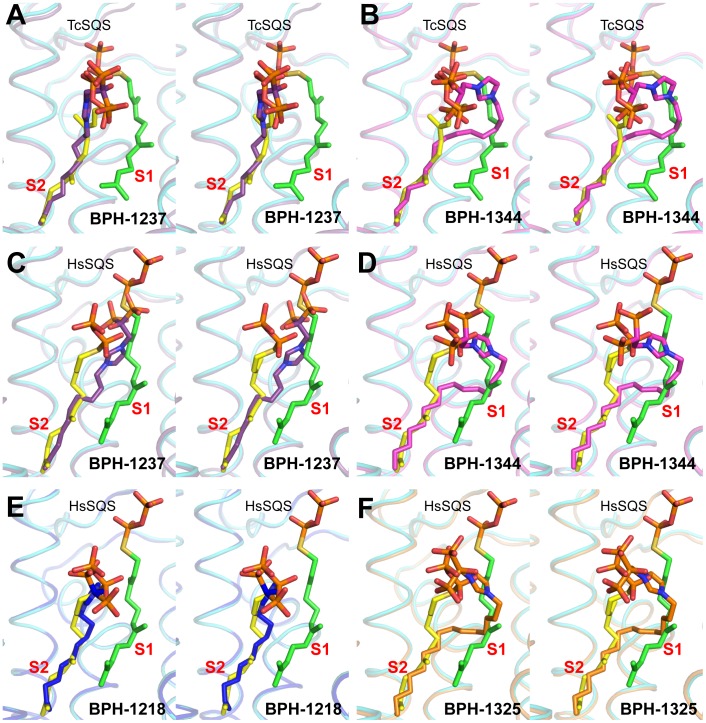
Complex Structures of SQS with lipophilic bisphosphonate inhibitors. (A) TcSQS/BPH-1237; (B) TcSQS/BPH-1344; (C) HsSQS/BPH-1237; (D) HsSQS/BPH-1344; (E) HsSQS/BPH-1218; (F) HsSQS/BPH-1325. All structures are superimposed on the corresponding FSPP structures (shown in green and yellow).

We next investigated the crystallographic structures of four representative lipophilic bisphosphonates (BPH-1218; BPH-1237; BPH-1325 and BPH-1344; Figure 1B) bound to TcSQS and/or HsSQS. These compounds contain C_10_ or C_15_ side-chains, with or without 1-OH groups on the bisphosphonate head-group (BPH-1218, C_10_, H; BPH-1237, C_10_, OH; BPH-1325, C_15_, OH; BPH-1344, C_15_, H). Data acquisition and structure refinement details are given in Tables 1 (for TcSQS) and 3 (for HsSQS). For TcSQS, the structure of the shorter-chain (C_10_) inhibitor (BPH-1237) is shown, superimposed on FSPP from the TcSQS/FSPP structure, in Figure 5A, and that of the longer chain (C_15_) inhibitor (BPH-1344) is shown superimposed on FSPP in Figure 5B. In both structures the S2 farnesyl-binding site is occupied by the n-alkyl side-chains, and the methyl terminus of each inhibitor reaches the same position, as do the terminal methyl groups in FSPP (as seen also with the quinuclidine inhibitors). With TcSQS, the electron density with the shorter chain inhibitor is well defined (Figure S2) and it is clear that the imidazolium and bisphosphonate groups occupy S2, Figure 5A. With the longer chain inhibitor (BPH-1344), the density is broken in several places, but the n-alkyl side chain spans both S1 and S2 sites, with strong bisphosphonate fragment density. We attribute this to the presence of multiple conformations in the middle part of the side chain.

For HsSQS, co-crystallizations with BPH-1218, BPH-1237, BPH-1325 and BPH-1344 were all successful and strong electron densities were obtained in all cases (Figure S2). With the shorter chain inhibitors, there are several ways in which the bisphosphonate head-groups bind (Figure 5C, E). With the des-hydroxy species BPH-1218, the inhibitor binds to the S2 site in a similar way to that observed in the TcSQS/BPH-1237 complex, with the polar head-group interacting with Arg52, Ser53, Ser51, Tyr73, Asn215 and Arg218. With the 1-OH bearing BPH-1237, the side-chain binds to the S2 site, but the head-group binds to the S1 site where it interacts with Arg77, Asp80 and Asp84 of the first DXXED motif. The imidazolium group of this S1-head/S2-tail BPH-1237 molecule thus occupies the same cationic binding site as seen with the quinuclidines. In the case of the two longer chain (C_15_) species bound to HsSQS (Figure 5D, F), the last 8 carbons in both chains locate to the S2 site, but the chain then bends and occupies the S1 site, basically in the “bend” region seen in CrtM/PSPP structures [27]. Then, the bisphosphonate polar head-group returns to the S2 region. This arrangement of the long alkyl chain presumably minimizes repulsive interactions with the protein since the binding energies are less than those with the C_10_ bisphosphonates, though as noted above, their improved hydrophobicity results in better cell growth inhibition. Detailed protein-ligand interactions for all 6 bisphosphonate structures are shown in Figure S5I–N.

Structure prediction algorithms such as Phyre 2 [41] predict that both *T. brucei* SQS as well as *Leishmania amazonensis* SQS (LmSQS) will have similar structures to *T. cruzi* SQS, making it likely that the new inhibitors described here will inhibit these enzymes as well. Moreover, in *T. brucei*, SQS has been validated as a drug target by use of RNA interference [42]. It has also been reported that LmSQS is potently inhibited by several quinuclidines including ER119884 and E5700, in the nanomolar/sub-nanomolar range, and these compounds are active against the parasites [43]. The selectivity of these drugs was demonstrated with the JC-1 mitochondrial fluorescent label and by trypan blue exclusion tests with macrophages, which showed that the IC_50_s against the host cells were 4 to 5 orders of magnitude greater that those against intracellular parasites [15], [40], [43].

### Structure of thiocyanate inhibitor WC-9 bound to human SQS

**Figure 6:**
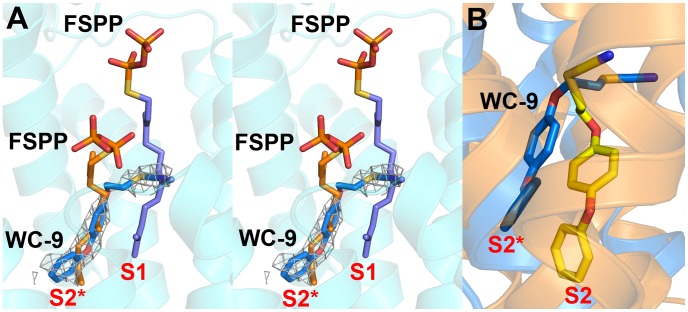
Structures of WC-9 bound to HsSQS and CrtM. (A) Stereo view of WC-9 bound to HsSQS. The 2F_o_ - F_c_ map is contoured at 1σ. (B) Structure of WC-9 bound to CrtM (yellow), overlaid with WC-9/HsSQS structure (blue).

In previous work [27] we found that there was close similarity between the structure of the ligand BPH-652 when bound to CrtM and HsSQS, in which the ligand binds primarily to S2 – a 2.0 Å RMSD for the BPH-652 ligand atoms when using the two aligned protein structures and only 0.6 Å for the two aligned ligands. We also reported the structure of the thiocyanate inhibitor WC-9 (Figure 1B) bound to CrtM and WC-9 is also known to be an inhibitor of TcSQS [17]. We were unable to obtain diffraction quality crystals of WC-9 bound to TcSQS, but we did obtain the crystal structure of WC-9 bound to HsSQS, as shown in Figure 6A. The ligand again appears to bind to S2 [44], however, when we compare the CrtM and HsSQS/WC-9 structures, Figure 6B, it is clear that there is a shift in the position of the S2 site (denoted S2* in Figure 6B).

### Comparisons between squalene and dehydrosqualene synthase structures

**Figure 7:**
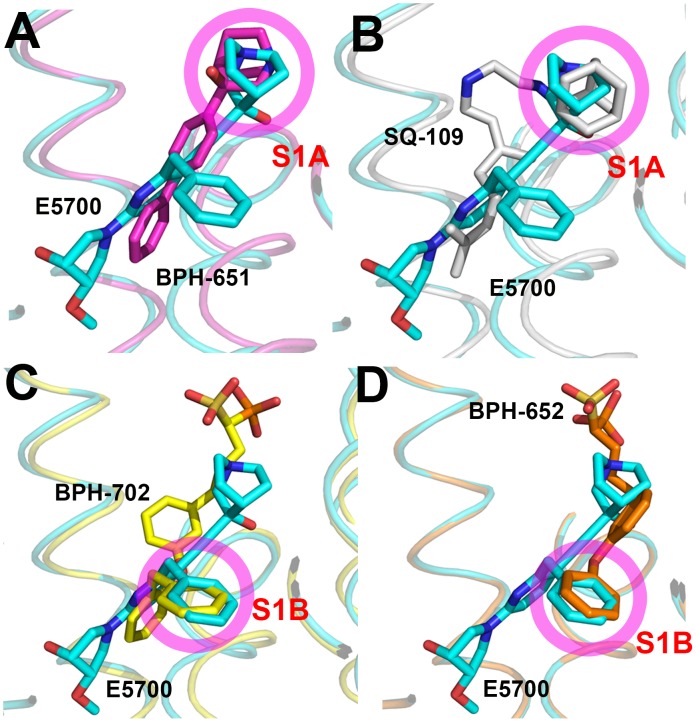
Comparisons between TcSQS/E5700 and CrtM/inhibitor structures. The CrtM inhibitors are: (A) BPH-651 (PDB ID code 3ACW). (B) SQ-109 (PDB ID code 4EA2). (C) BPH-702 (PDB ID code 3ACY). (D) BPH-652 (PDB ID code 2ZCQ).

The results described above are also of interest when compared with earlier results on the bacterial dehydrosqualene synthase (CrtM, from *Staphylococcus aureus*) since they highlight two similar inhibitor structural features. The first observation is that the quinuclidine ring in E5700 as well as in ER119884 occupies a similar (S1A) binding site to that seen in the SQS/CrtM quinuclidine inhibitor BPH-651 (Figure 1B) bound to CrtM (Figure 7A). This site is also occupied by the adamantane ring in the CrtM inhibitor [44] SQ-109 (Figures 1B and 7B) and this site represents a common lipophilic, cationic binding site. The second observation of interest is that there is a hydrophobic pocket (S1B) that is occupied by the benzyl group in E5700 bound to SQS, as well as to the benzyl group in BPH-702 (Figures 1B and 7C) or the benzyl group in BPH-652 (Figures 1B and 7D). These observations suggest that it may be possible to improve the activity of other inhibitors (such as the bisphosphonates) by incorporating benzyl groups that can bind to S1B.

### Synergy between E5700 and the lanosterol 14α-demethylase inhibitor posaconazole

The results described above provide information on the molecular basis for the potency of the aryl-quinuclidines as SQS inhibitors and potential future strategies for the design of parasite-specific inhibitors. The potency of these compounds might also be enhanced by using them in combination with other inhibitors, acting at different steps in the ergosterol biosynthesis pathway [45]. We tested this idea using a combination of E5700 and posaconazole (a very potent lanosterol 14α-demethylase (P450) inhibitor in clinical trials for Chagas disease) on intracellular amastigotes cultured inside murine peritoneal macrophages using the methodology described previously to investigate the combined effects of amiodarone and posaconazole [46]. As can be seen in Figure 4D, we obtain a concave isobologram with a fractional inhibitory concentration index (FICI) of 0.36, which indicates synergism since the accepted value for synergistic effects is FICI<0.5 [47].

In summary, in this work we report the structure of squalene synthase from *Trypanosoma cruzi*, a potentially important target for the development of novel therapeutic agents for the treatment of Chagas disease. The SQS protein structure had two major hydrophobic pockets: The S1 allylic binding site in which FSPP(FPP) binds to a cluster of highly conserved Asp residues via Mg^2+^, and a homoallylic S2 binding site to which the second FPP binds via basic residues interacting with the FSPP's diphosphate group. We solved the structures of two potent SQS inhibitors, the quinuclidines E5700 and ER119884, bound to both TcSQS as well as HsSQS. The results show that in all four cases, the more distal parts of the hydrophobic side-chains occupy primarily the S2-like FPP-binding site, while the benzyl groups bind to S1B and occupy the same position as the terminal methyl groups in the S1 FPP. The quinuclidinol groups occupy the S1A site where cationic species have been found to bind in the bacterial SQS homolog, CrtM. There were, however, several differences in inhibitor as well as amino-acid side-chain structures between TcSQS and HsSQS, opening up the possibility of selective inhibitor design. We also screened a library of lipophilic bisphosphonates against TcSQS, HsSQS, TcFPPS and *T. cruzi* amastigote cell growth. Most potent inhibition was found against TcSQS with a C_11_ side-chain, but the most potent activity in cells was with a C_15_ compound, due we propose to its enhanced hydrophobicity and hence, better cell penetration. There was no or little rescue from cell growth inhibition by farnesol or geranylgeraniol, but ergosterol biosynthesis, essential for parasite survival, was inhibited, consistent with an SQS target. We then determined the structures of four bisphosphonates bound to HsSQS and two of these bound to TcSQS. The results showed that in all cases ∼8 carbons at the chain terminus bound to S2 and were closely superposed on the more distal parts of the FSPP binding sites while the imidazolium rings bound close to the cationic binding sites identified earlier in CrtM. With the longer chain compounds, the upper part of the n-alkyl side-chain (close to the bisphosphonate head group) occupied the S1A pocket that is occupied by the “kinked” part of the FSPP structure, seen also in CrtM (+FSPP or PSPP) structures [27]. The polar head-groups bound primarily to the S2 head-group region. Both the bisphosphonates as well as the quinuclidine inhibitors thus bind to both the S1 and S2 substrate-binding sites, providing potent inhibition, leading to strong antiparasite activity. We also found strong synergy between the quinuclidine E5700 and and the 14α-demethylase inhibitor posaconazole, suggesting new therapeutic possibilities. Overall, the results presented here are of interest in the context of the future development of squalene synthase inhibitors as anti-infective drug leads against diseases caused by trypanosomatid parasites – the neglected tropical diseases.

## Materials and Methods

### Protein expression and purification

The constructs of TcSQS and HsSQS were obtained as described previously [16], [30]. TcSQS expression was induced with 0.8 mM isopropyl β-thiogalactopyranoside (IPTG) at 25°C for 18 hours, and HsSQS with 1.0 mM IPTG at 37°C for 6 hours. Cell pastes were harvested by centrifugation at 4,000× *g* for 15 min and resuspended in a lysis buffer containing 25 mM Tris-HCl, pH 7.5, 20 mM imidazole, and 150 mM NaCl. Cell lysates were prepared with a French pressure cell press (AIM-AMINCO Spectronic Instruments), and then centrifuged at 17,000× *g* for 30 min to remove cell debris. The cell-free extract was loaded onto lysis buffer-equilibrated Ni-NTA columns, followed by washing with 20 mM imidazole-containing buffer. The His-tagged enzymes were then eluted with an imidazole gradient from 20–250 mM, dialyzed twice against 5 L of 25 mM Tris-HCl, pH 7.5, and loaded onto a 20 ml DEAE Sepharose Fast Flow column (GE Healthcare Life Sciences). The buffer and gradient were 25 mM Tris, pH 7.5, and 0–500 mM NaCl. Purity of the recombinant proteins (>95%) was checked by SDS-PAGE analysis.

### Inhibition of SQS, FPPS and SPPS activity

Initial SQS enzyme inhibition assays were carried out, in duplicate, in 96-well plates, with a total of 200 µL of reaction mixture in each well. Reactions were monitored by using a continuous spectrophotometric assay for phosphate releasing enzymes [26]. Reaction buffer contained 50 mM Tris-HCl, 1 mM MgCl_2_, 450 µM FPP, pH 7.4. The compounds investigated were pre-incubated with 2 µg of SQS for 30 min at 20°C. The IC_50_ values were obtained by fitting the inhibition data to a normal dose–response curve, using GraphPad PRISM, version 4.0, software for Windows (GraphPad Software Inc., San Diego, CA, www.graphpad.com). We then verified the results by using a radioactivity assay using ^3^H-labelled FPP as substrate. The reaction mixture (50 µL) had 2.5 nM *T. cruzi* or *H. sapiens* SQS, 0.5 µM [1-^3^H]-FPP, and 250 µM NADPH, in a detergent-containing buffer (50 mM HEPES-KOH, pH 7.5, 100 mM NaCl, 4 mM MgCl_2_, 0.5% Tween 80, 2 mM CHAPS, and 4 mM DTT). The enzymes were incubated with inhibitor for 10 minutes before addition of the substrates. The reaction was allowed to continue at 37°C for 15 minutes and then terminated with 50 µL of 0.1 M NaOH/methanol. The product was extracted with 500 µL of hexanes and 300 µL of the organic layer was measured by scintillation counting.

For FPPS inhibition we used the methods described previously [22]. The condensation of geranyl diphosphate (100 µM) and isopentenyl diphosphate (100 µM) was monitored at room temperature by using a continuous spectrophotometric assay for phosphate releasing enzymes [48]. The reaction buffer contained 50 mM Tris-HCl, pH 7.4, 1 mM MgCl_2_, and 0.01% Triton X100. The compounds investigated were pre-incubated with enzyme for 30 min at room temperature. The IC_50_ values were obtained from fitting dose-response curve using Prism 4.0 (GraphPad Software, Inc., La Jolla, CA, www.graphpad.com).

The activity of *T. cruzi* SPPS was determined by a radiometric assay [37]. Briefly, 100 µL of assay buffer (100 mM Tris–HCl buffer, pH 7.4, 1 mM MgCl_2_, 1% (v/v) Triton X-100, 7.07 µM [4-^14^C]-IPP (10 µCi/µmol)), and 50 µM GGPP was pre-warmed to 37°C. The assay was initiated by the addition of 10–20 ng of recombinant protein. The assay was allowed to proceed for 30 min at 37°C and was quenched by chilling quickly in an ice bath. The reaction products were extracted with 1 mL of 1-butanol saturated with water. The organic layer was washed with water saturated with NaCl, and transferred to a scintillation vial with 4 mL of Ecolume scintillation solution for counting. One unit of enzyme activity was defined as the activity required to incorporate 1 nmol of [4-^14^C]-IPP into [4-^14^C]-FPP in 1 min.

### *T. cruzi* cell growth inhibition

Gamma-irradiated (2,000 Rads) Vero cells (3.4×10^4^ cells/well) were seeded in 96 well plates (black, clear bottom plates from Greiner Bio-One) in 100 µL RPMI media (Sigma) with 10% FBS. Plates were incubated overnight at 35°C and 7% CO_2_. After overnight incubation, Vero cells were challenged with 3.4×10^5^ trypomastigotes/well (CL strain overexpressing a tdTomato red fluorescent protein) in 50 µL volume and incubated for 5 h at 35°C and 7% CO_2_. After infection, cells were washed once with Hanks solution (150 µL/well) to eliminate any extracellular parasites, and compounds were added in serial dilutions in RPMI media in 150 µL volumes. Each dilution was tested in quadruplicate. Each plate also contained controls with host cells and no parasites (for a background check), and controls with parasites and no drugs (positive control). Drugs were tested at 5 different concentrations (highest 25 µM). For each set of experiments, benznidazole was used as a positive control. After drug addition, plates were incubated at 35°C and 7% CO_2_. At day 3 post-infection, plates were assayed for fluorescence and IC_50_ values were determined by non-linear regression analysis using SigmaPlot.

### Sterol biosynthesis inhibition

*T. cruzi* epimastigotes were grown for 72 hours at 28°C in liver infusion tryptose medium [49] supplemented with 10% heat inactivated fetal bovine serum in the absence or presence of 2 µM BPH-1218. Cells from cultures (96 h incubation) were washed with Buffer A with glucose (116 mM NaCl, 5.4 mM KCl, 0.8 mM MgSO_4_, 5.5 mM D-glucose, and 50 mM HEPES at pH 7.0) then pelleted and chloroform/methanol (2∶1 v/v) added (∼30 mL of solvent per gram of wet cells) followed by vigorous stirring and overnight incubation at 4°C. The extract was then filtered using Whatman #2 paper to eliminate insoluble material, and the filtrate (a single phase) dried under nitrogen. The extract was then re-extracted using chloroform/methanol 9/1 v/v, dried under nitrogen, dissolved in 1 mL of pure chloroform and re-dried under nitrogen gas. The extracted lipids were saponified with 5 mL of ethanolic-potassium hydroxide (5 g KOH, 7 mL water, 14 mL ethanol) for 1 hour at 80°C. After cooling, 2.5 mL of water and 1.5 mL hexane were added and the phases allowed to separate. The aqueous phase was then re-extracted twice with hexane. The hexane extracts were dried under nitrogen, dissolved in chloroform and converted to the trimethylsilyl derivatives by using bis-trimethylsilyl amide. Both standards and samples (1 µL) were injected in split mode (10∶1) on a GC/MS system, which consisted of an Agilent 6890 gas chromatograph, an Agilent 5973 mass selective detector and an HP 7683B (Agilent Inc, Palo Alto, CA, USA) auto-sampler. Samples were analyzed on a 30 m DB5 column with 0.32 mm I.D. and 0.25 µm film thickness (Agilent Inc, Palo Alto, CA, USA) with an injection port temperature of 300°C, the interface was set to 300°C, the ion source adjusted to 230°C, MS Quadrupole 150°C. The helium carrier gas was set at a constant flow rate of 2.7 mL min^−1^. The temperature program was: 2 min at 250°C, followed by an oven temperature increase of 25°C min^−1^ to 320°C, for 10 min. The mass spectrometer was operated in positive electron impact mode (EI) at a 69.9 eV ionization energy in the m/z 50–800 scan range. The spectra of all chromatogram peaks were evaluated by using the HP Chemstation (Agilent, Palo Alto, CA, USA) program. Identifications and quantifications were performed using mass spectra obtained from authentic standards and with NIST08 and W8N08 libraries (John Wiley & Sons, Inc., USA).

### Quinone extraction and analysis

For ubiquinone extraction, epimastigotes (1 g wet weight) were washed twice with buffer A with glucose (116 mM NaCl, 5.4 mM KCl, 0.8 mM MgSO_4_, 5.5 mM D-glucose, and 50 mM HEPES at pH 7.0). To break the cells, the pellets were frozen in a dry ice-100% ethanol bath (for 7 min) and thawed at 37°C (for 3 min) for a total of 5 times. One volume of water and three volumes of 1-propanol were added to the pellet. The samples were vortexed for 30 sec at room temperature and allowed to stand for one minute. Two volumes of n-hexane were added, the mixture vortexed for 30 sec at room temperature, then centrifuged at 12,000× g for 5 min. The upper phase, containing ubiquinone, was collected into a glass tube, and dried under nitrogen gas, then analyzed using LC-MS.

LC/MS was performed using an Agilent LC/MSD Trap XCT Plus system (Agilent Technologies, Santa Clara, CA) with an 1100 series HPLC system including a degasser, an auto-sampler, a binary pump, and a multiple-wavelength detector. The LC separation used a Phenomenex Gemini 3u C18 110A column (2 mm×100 mm) (Torrence, CA) with mobile phase A (0.1% formic acid in water) and mobile phase B (0.1% formic acid in acetonitrile). The flow-rate was 0.25 mL/min. The linear gradient was as follows: 0–1 min, 100% A; 7–50 min, 0% A; 51–56 min, 100% A. The auto-sampler was set at 5°C and the injection volume was 15 µL. UV signals were recorded at 275 nm. Positive ion mass spectrometry used atmospheric pressure chemical ionization (APCI). The capillary voltage was 4000 V and the nebulizer pressure and drying gas flow were 60 psi, 7 L/min, respectively. Temperatures for drying gas and APCI Vap were 350°C and 400°C, respectively.

### Isothermal titration calorimetry (ITC)

ITC experiments were carried out basically as described previously for ligands binding to FPPS [50]. The thermodynamic parameters were: *ΔG* = −32.1 kJ·mol^−1^, *ΔH* = −10.4 kJ·mol^−1^, *ΔS* = 74.3 J·mol^−1^·K^−1^, and *n* = 1.86.

### Cell growth inhibition by E5700+ posaconazole

The method used to determine possible synergism between the quinuclidine E5700 and the azole posaconazole followed that described by Veiga-Santos et al [46].

### Measuring Vero cell proliferation using alamarBlueTM

Vero cells (1.7×10^4^ cells/well) were seeded in 96 well plates (black, clear bottom from Greiner Bio-One Cat#655090) in 100 µL RPMI media (Sigma Cat# R-4130) with 10% FBS. Plates were tested as described by Recher et al [51].

### Protein crystallization, data collection and structure determination

Initial crystallization screening was performed using Hampton Research Crystal Screens (Laguna Niguel, CA, USA) with the hanging-drop vapor-diffusion method at room temperature. In general, 2 µL TcSQS protein solution (8 mg/mL TcSQS enzyme, 25 mM Tris-HCl, pH 7.5, 2.5 mM FSPP) was mixed with 2 µL of reservoir solution. Single crystals were obtained in 0.1 M Tris, pH 8.5, 21% PEG 3350. The crystals were mounted in a cryo-loop and flash-frozen in liquid nitrogen with 0.15 M Tris, pH 8.5, 25% PEG3350 and 8% glycerol as a cryo-protectant. For HsSQS, 2 µL protein solution (5 mg/mL HsSQS enzyme, 25 mM Tris-HCl, pH 7.5) was mixed with 2 µL of reservoir solution. Single crystals were obtained in 0.2 M sodium citrate, pH 8.2, 28% PEG 3350. Prior to data collection at 100 K, the crystals were mounted in a cryo-loop and flash-frozen in liquid nitrogen with 0.3 M sodium citrate, pH 8.2, 30% PEG3350 and 2% glycerol as a cryo-protectant.

Crystals of TcSQS could only be obtained in the presence of FSPP, and direct co-crystallization of TcSQS with ER119884 and E5700 was not successful. Instead, we soaked the crystals of the TcSQS-FSPP complex with cryo-protectant solution containing 10 mM ER119884 or E5700 for at least 4 hrs prior to data acquisition. Crystals of (human) HsSQS in complex with the inhibitors FSPP, ER119884, E5700, BPH-1218, BPH-1237 and BPH-1325 were obtained by co-crystallization under similar conditions (0.2 M sodium citrate, pH 8.2, 24–28% PEG 3350, 5 mM inhibitor concentration).

Diffraction data were collected at beam lines BL13B1 and BL13C1 of the National Synchrotron Radiation Research Center (NSRRC, Hsinchu, Taiwan), and processed using the program HKL2000. Prior to structure refinement, 5% randomly selected reflections were set aside for calculating R_free_ as a monitor of model quality. The crystal structure of TcSQS was solved by using the molecular replacement (MR) method using the CNS program [52]. The previously determined HsSQS structure (PDB code 3VJA) was used as a search model (since it has 44% identity to TcSQS). The TcSQS crystal belongs to the space group P2_1_2_1_2_1_ and contains four protein molecules in an asymmetric unit. The other TcSQS-drug complex structures were solved by using the refined TcSQS (but without the bound FSPP) as a starting model. All crystals of HsSQS belong to the space group P2_1_ and contain six protein molecules in the asymmetric unit. NCS restraints were applied at early stages of refinement and released at later stages. The refinement of every structure started with a temperature factor (B-factor) of 30, but upon rigid-body refinement an overall B was calculated for best scaling, which should be close to the corresponding Wilson B-factor. The individual atomic B-factors were not restrained by NCS and average B values are shown for the protein, ligand and solvent atoms in Tables 1–3. The rmsd between the four monomers of TcSQS ranged from 0.34 to 0.68 Å for 341 Cα atoms. The rmsd between six HsSQS monomers were 0.23–0.80 Å for 324–335 Cα atoms. The rmsd between TcSQS and HsSQS were larger, ranging from 0.82 to 1.28 Å for 264–273 Cα atoms.

For all drug molecules, the descriptions of atoms, structures, chemical bonds and their restraints were compiled into respective “topology” and “parameter” files by using the PRODRG server [53] for structural refinement by using CNS. Manual adjustments of the models and addition of water molecules used the program COOT [54].

### Accession numbers

The atomic coordinates and structure factors have been deposited in the RCSB Protein Data Bank, www.pdb.org for *T. cruzi* SQS complexes with FSPP (3WCA), E5700 (3WCC), ER119884 (3WCE), BPH-1237 (3WCB) and BPH-1344 (3WCG), and human SQS complexes with FSPP (3WC9), E5700 (3WCJ), ER119884 (3WCM), BPH-1218 (3WCF), BPH-1237 (3WCH), BPH-1325 (3WCI), BPH-1344 (3WCL) and WC-9(3WCD).

## Supporting Information

Figure S1 **Schematic illustration of FPP-to-squalene reaction catalyzed by SQS.** The mechanism is based on the CrtM crystallographic results by Lin et al. [27]. (A) Bound FPP in S1 ionizes. (B) Carbocation reacts with S2 FPP alkene group. (C) PSPP PPi in S2 moves to S1 site. (D) PSPP PPi ionizes. (E and F) Carbocation rearranges and is reduced by NADPH to form the final product, squalene. (G) Alternative view of the reaction mechanism.
(TIF)

Figure S2 **Electron density maps of TcSQS and HsSQS bound ligands.** Electron density maps in red and brown represent 3σ and 1σ, respectively. (A) to (E) The Fo-Fc omit maps of FsPP, E5700, ER119884, BPH-1237 and BPH-1344 from TcSQS complex structures and colored in cyan, green, magenta, blue and yellow, respectively. (F) to (L) The Fo-Fc omit maps of FsPP, E5700, ER119884, BPH-1344, BPH-1237, BPH-1325 and BPH-1218 from HsSQS complex structures and colored in cyan, green, magenta, blue, yellow, gray and purple, respectively.
(TIF)

Figure S3 **Ligand electron densities in TcSQS for FSPP, E5700 and ER119884.** The refined models are superimposed on Fo-Fc difference Fourier maps calculated by omitting the ligands in question. Each ligand is shown as a stick model with green carbon atoms. The “OMIT” maps are all contoured at 3.0 σ level and shown as purple mesh representations. (A) TcSQS/FSPP (PDB ID code 3WCA); (B) TcSQS/E5700 (PDB ID code 3WCC); (C) TcSQS/ER119884 (PDB ID code 3WCE).
(TIF)

Figure S4 **Isothermal titration calorimetry results for FSPP binding to TcSQS.**
(TIF)

Figure S5 **Ligplot representations of local interactions.** (A) TcSQS+FSPP, site 1; (B) TcSQS+FSPP, site 2; (C) HsSQS+FSPP, site 1; (D) HsSQS+FSPP, site 2; (E) TcSQS+E5700; (F) TcSQS+ER119884; (G) HsSQS+E5700; (H) HsSQS+ER119884; (I) TcSQS+BPH-1237; (J) TcSQS+BPH-1344; (K) HsSQS+BPH-1218; (L) HsSQS+BPH-1237; (M) HsSQS+BPH-1325; (N) HsSQS+BPH-1344.
(PDF)

Figure S6 **Ligand electron densities in HsSQS for FSPP, E5700 and ER119884.** The Fo-Fc “OMIT” maps are contoured at the 3.0 σ level. (A) HsSQS/FSPP (PDB ID code 3WC9); (B)HsSQS/E5700 (PDB ID code 3WCJ); (C) HsSQS/ER119884 (PDB ID code 3WCM).
(TIF)

Figure S7 **Stereo-view of electron densities of quinuclidines bound to TcSQS.** (A) E5700 and (B) ER119884. The Fo-Fc “OMIT” maps are contoured at 3.0 σ level. The corresponding models are superimposed and shown as cartoon for the protein and sticks for the ligand, with the carbon atoms colored in green. For comparison, the aligned structure of TcSQS/FsPP is also shown, as cartoon and sticks with cyan carbons.
(TIF)

Figure S8 **Effects of BPH-1218 on ergosterol and ubiquinone biosynthesis in** ***T. cruzi*** **epimastigotes.** (A) GC-MS trace for trimethylsilyated sterols and sterol compositions (+drug %; −drug % and +drug/−drug ratios). (B)–(E) LC-MS and LC-UV results for UQ_9_ biosynthesis inhibition. (B, C). LC-MS without (B) or with (C) BPH-1218 in the growth medium. (D, E) LC-UV without (D) or with (E) BPH-1218 in the growth medium.
(TIF)
