# Correction: Squalene Synthase As a Target for Chagas Disease Therapeutics

**DOI:** 10.1371/journal.ppat.1004239

**Published:** 2014-06-10

**Authors:** The PLOS Pathogens Staff

The affiliation for the authors Na Shang, Qian Li, and Tzu-Ping Ko are incorrect. Na Shang and Qian Li are affiliated only with Tianjin Institute of Industrial Biotechnology, Chinese Academy of Sciences, Tianjin, China. Tzu-Ping Ko is affiliated only with the Institute of Biological Chemistry, Academia Sinica, Taipei, Taiwan.
