# Supplementary figures and images for: Squalene Synthase As a Target for Chagas Disease Therapeutics
Source: PLoS Pathog. 2014 May 1;10(5):e1004114. doi: 10.1371/journal.ppat.1004114 (PMC4006925; doi:10.1371/journal.ppat.1004114)

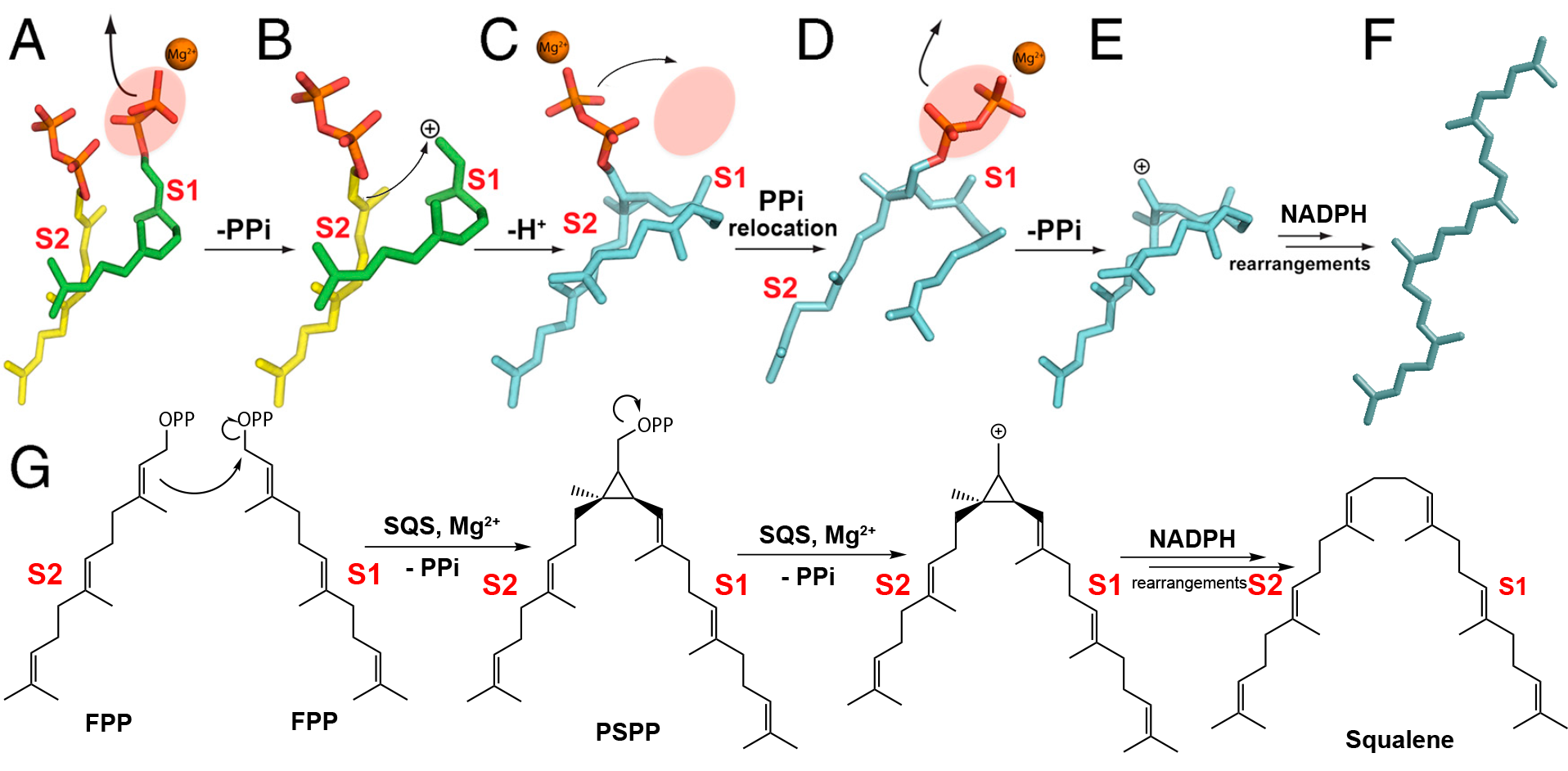

Supplement: Figure S1 — Schematic illustration of FPP-to-squalene reaction catalyzed by SQS. The mechanism is based on the CrtM crystallographic results by Lin et al. [27]. (A) Bound FPP in S1 ionizes. (B) Carbocation reacts with S2 FPP alkene group. (C) PSPP PPi in S2 moves to S1 site. (D) PSPP PPi ionizes. (E and F) Carbocation rearranges and is reduced by NADPH to form the final product, squalene. (G) Alternative view of the reaction mechanism. (TIF) [file ppat.1004114.s001.tif]

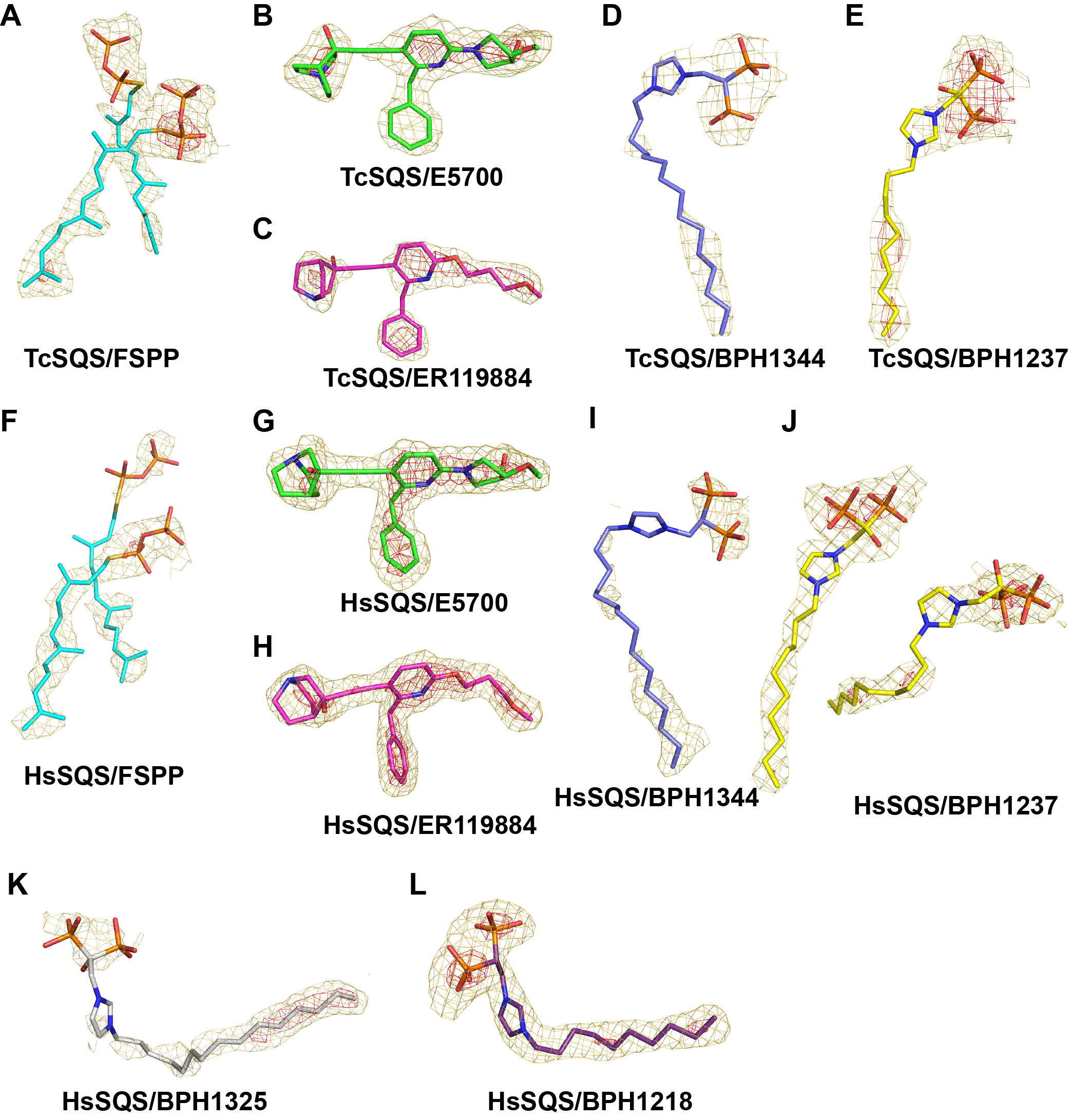

Supplement: Figure S2 — Electron density maps of TcSQS and HsSQS bound ligands. Electron density maps in red and brown represent 3σ and 1σ, respectively. (A) to (E) The Fo-Fc omit maps of FsPP, E5700, ER119884, BPH-1237 and BPH-1344 from TcSQS complex structures and colored in cyan, green, magenta, blue and yellow, respectively. (F) to (L) The Fo-Fc omit maps of FsPP, E5700, ER119884, BPH-1344, BPH-1237, BPH-1325 and BPH-1218 from HsSQS complex structures and colored in cyan, green, magenta, blue, yellow, gray and purple, respectively. (TIF) [file ppat.1004114.s002.tif]

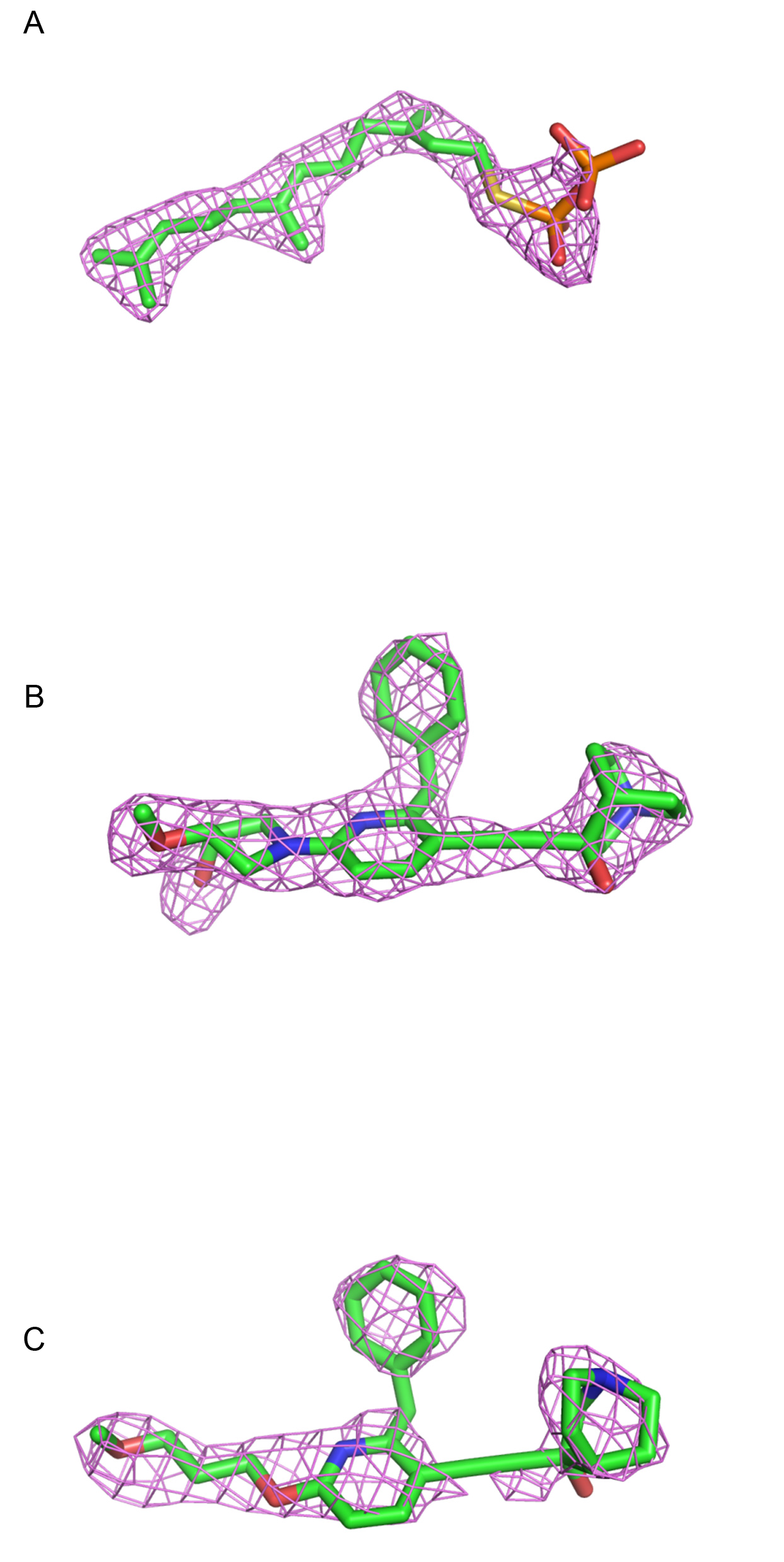

Supplement: Figure S3 — Ligand electron densities in TcSQS for FSPP, E5700 and ER119884. The refined models are superimposed on Fo-Fc difference Fourier maps calculated by omitting the ligands in question. Each ligand is shown as a stick model with green carbon atoms. The “OMIT” maps are all contoured at 3.0 σ level and shown as purple mesh representations. (A) TcSQS/FSPP (PDB ID code 3WCA); (B) TcSQS/E5700 (PDB ID code 3WCC); (C) TcSQS/ER119884 (PDB ID code 3WCE). (TIF) [file ppat.1004114.s003.tif]

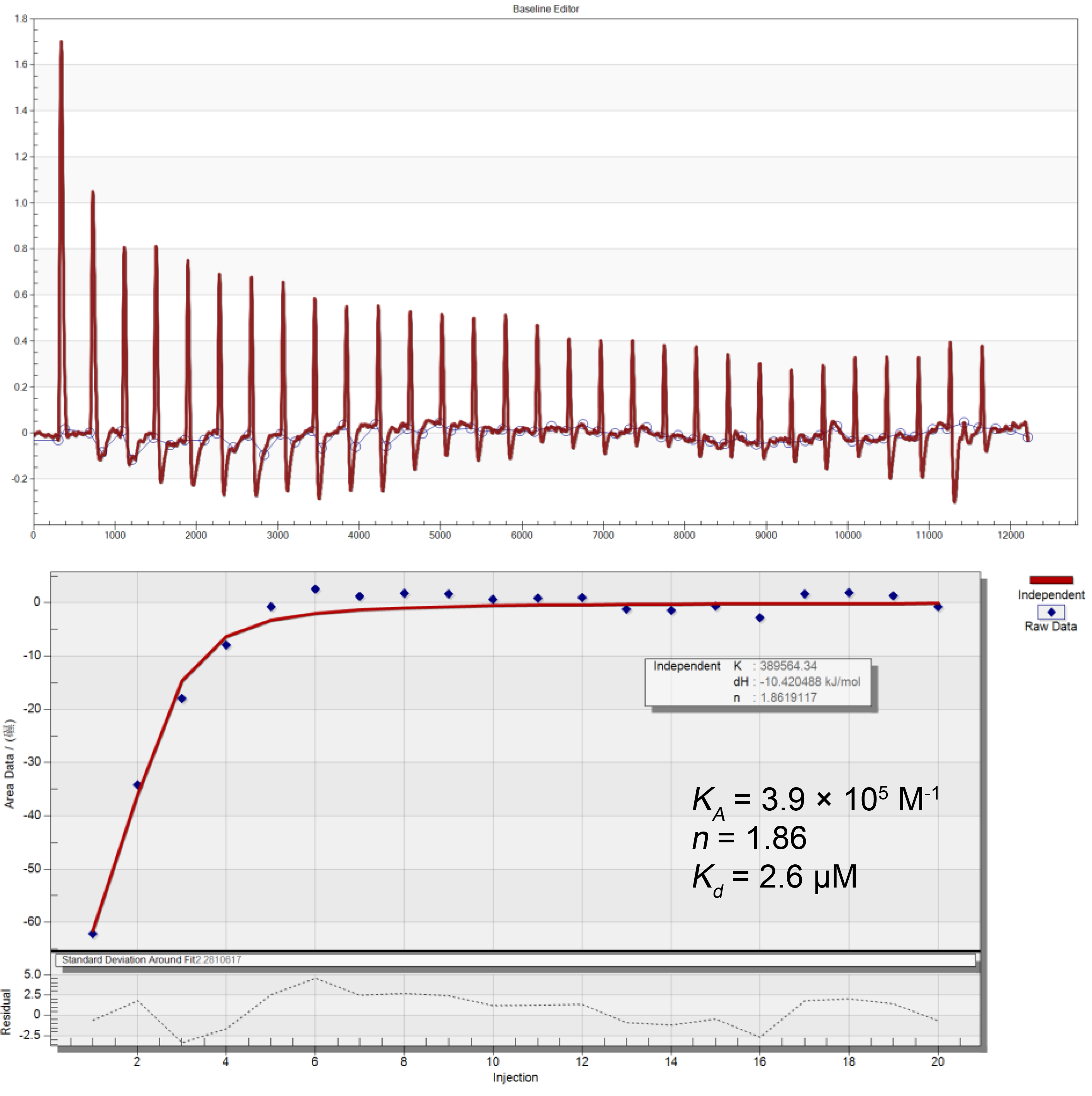

Supplement: Figure S4 — Isothermal titration calorimetry results for FSPP binding to TcSQS. (TIF) [file ppat.1004114.s004.tif]

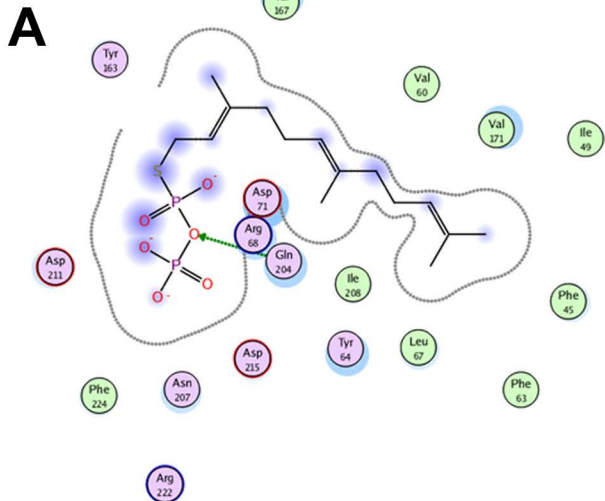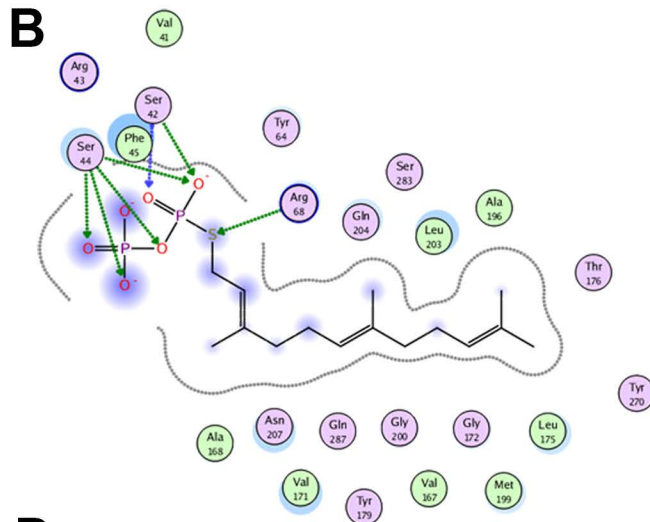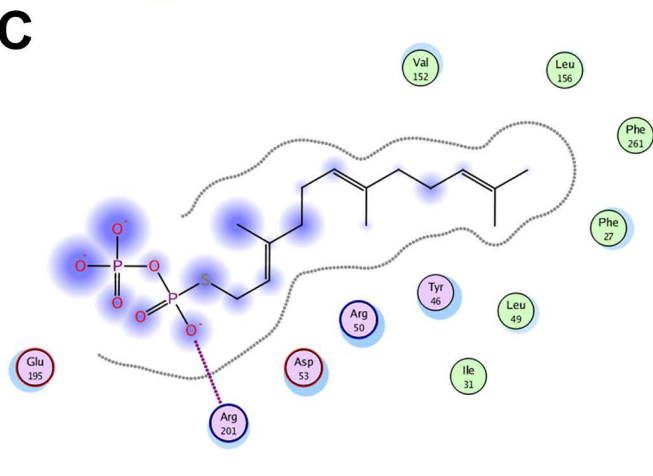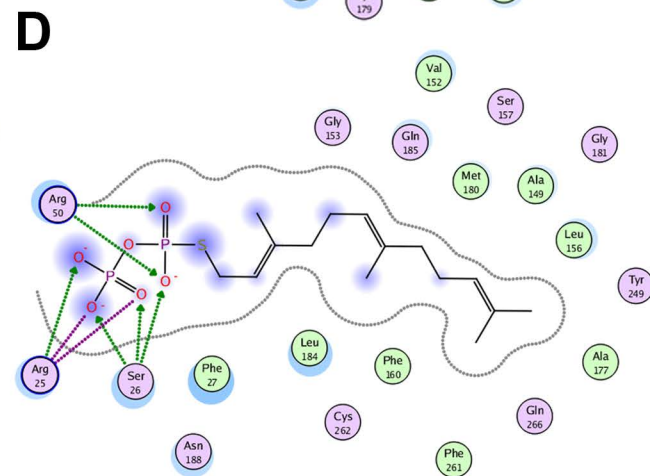

**E**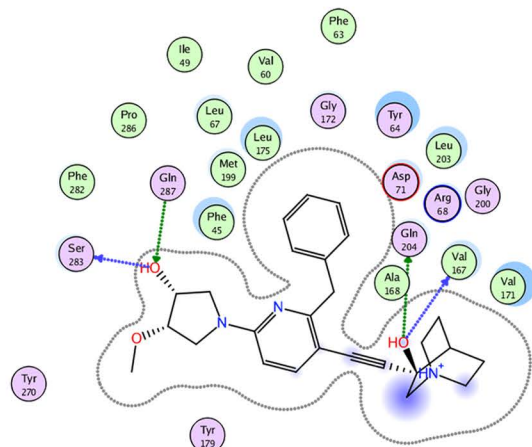**F**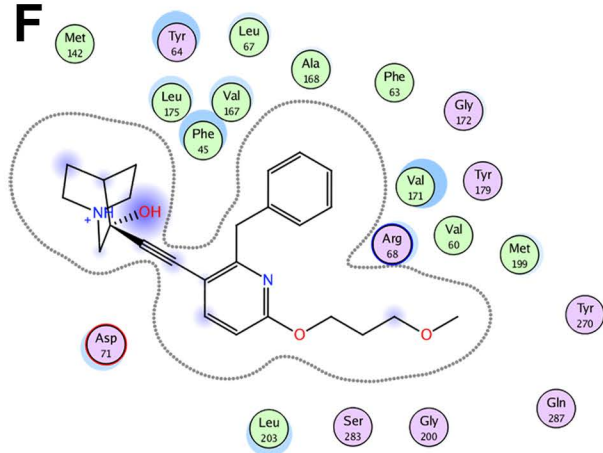**G**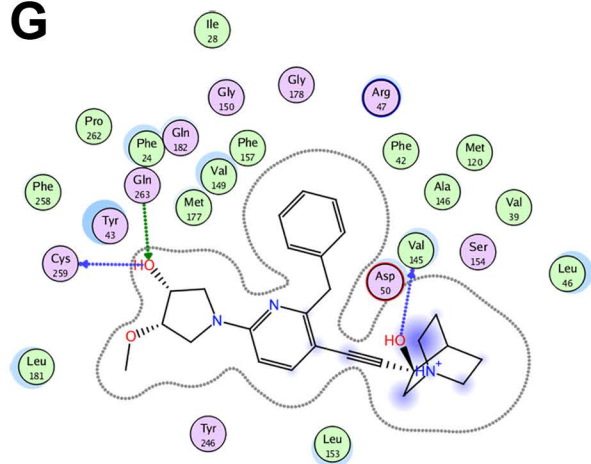**H**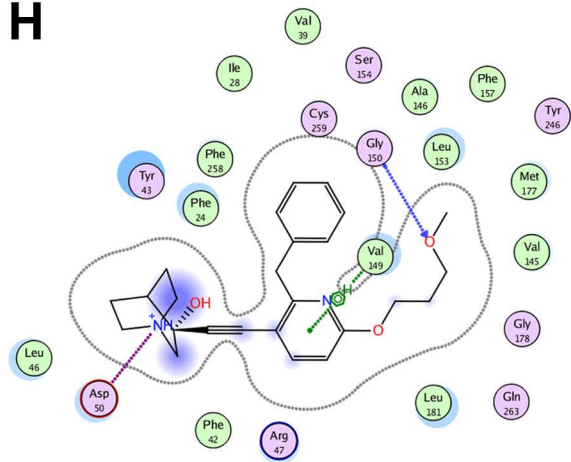**I**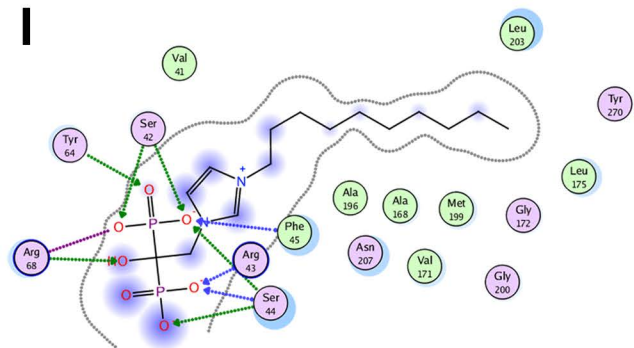**J**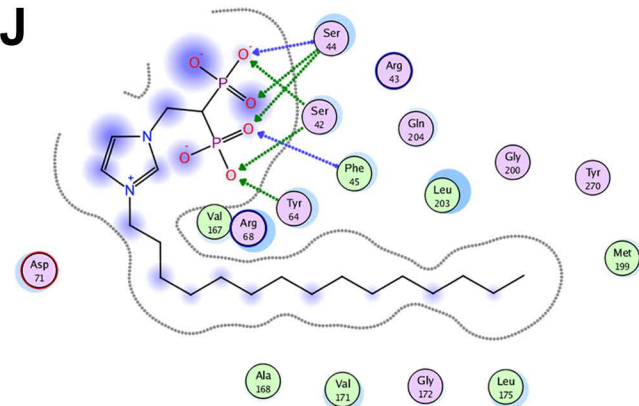

**K**

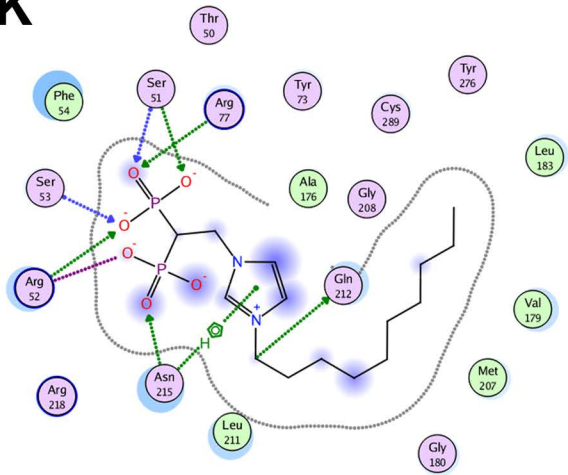

**L**

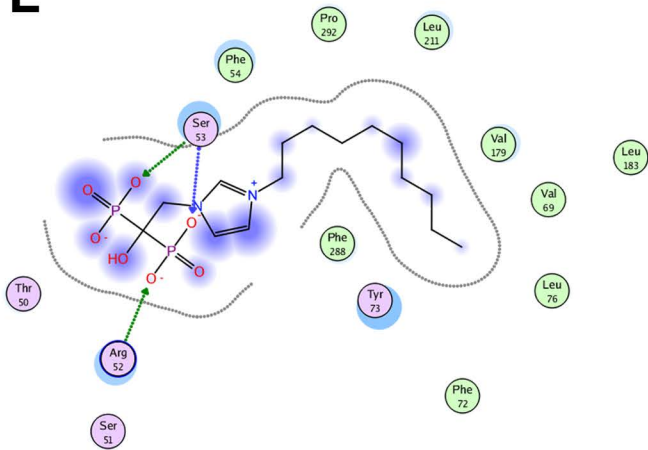

# M

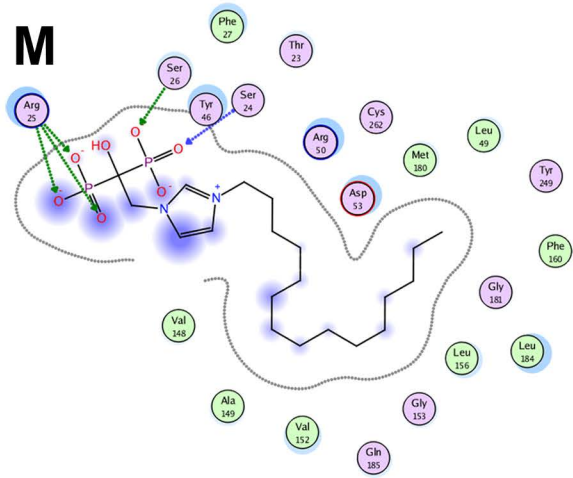

N

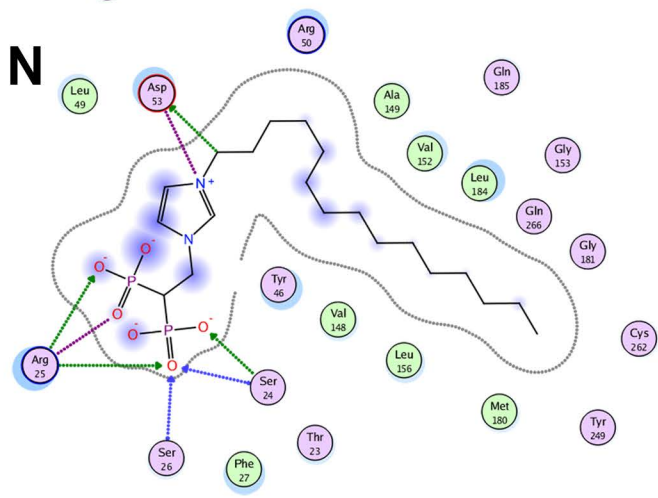

Supplement: Figure S5 — Ligplot representations of local interactions. (A) TcSQS+FSPP, site 1; (B) TcSQS+FSPP, site 2; (C) HsSQS+FSPP, site 1; (D) HsSQS+FSPP, site 2; (E) TcSQS+E5700; (F) TcSQS+ER119884; (G) HsSQS+E5700; (H) HsSQS+ER119884; (I) TcSQS+BPH-1237; (J) TcSQS+BPH-1344; (K) HsSQS+BPH-1218; (L) HsSQS+BPH-1237; (M) HsSQS+BPH-1325; (N) HsSQS+BPH-1344. (PDF) [file ppat.1004114.s005.pdf]

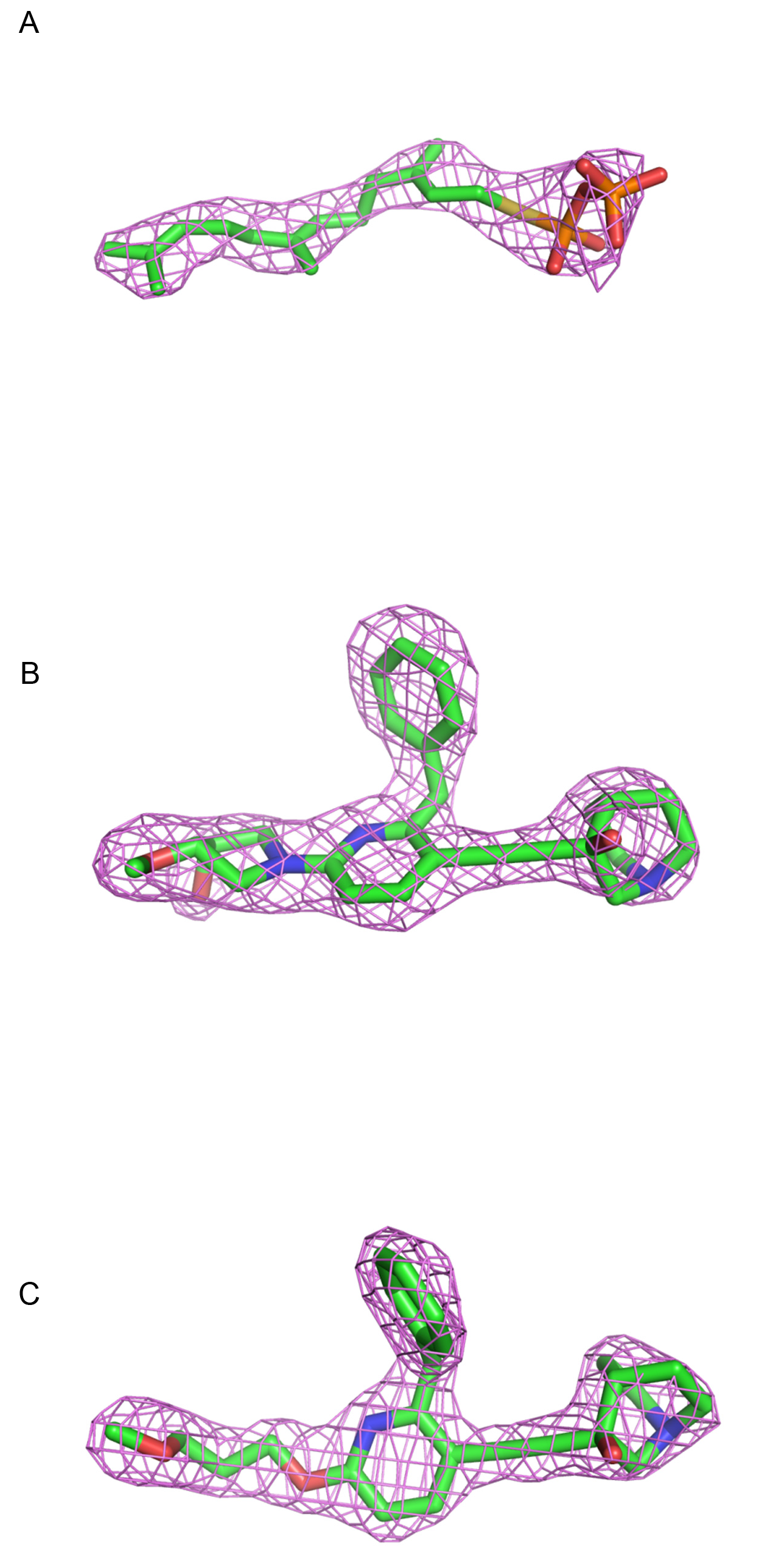

Supplement: Figure S6 — Ligand electron densities in HsSQS for FSPP, E5700 and ER119884. The Fo-Fc “OMIT” maps are contoured at the 3.0 σ level. (A) HsSQS/FSPP (PDB ID code 3WC9); (B)HsSQS/E5700 (PDB ID code 3WCJ); (C) HsSQS/ER119884 (PDB ID code 3WCM). (TIF) [file ppat.1004114.s006.tif]

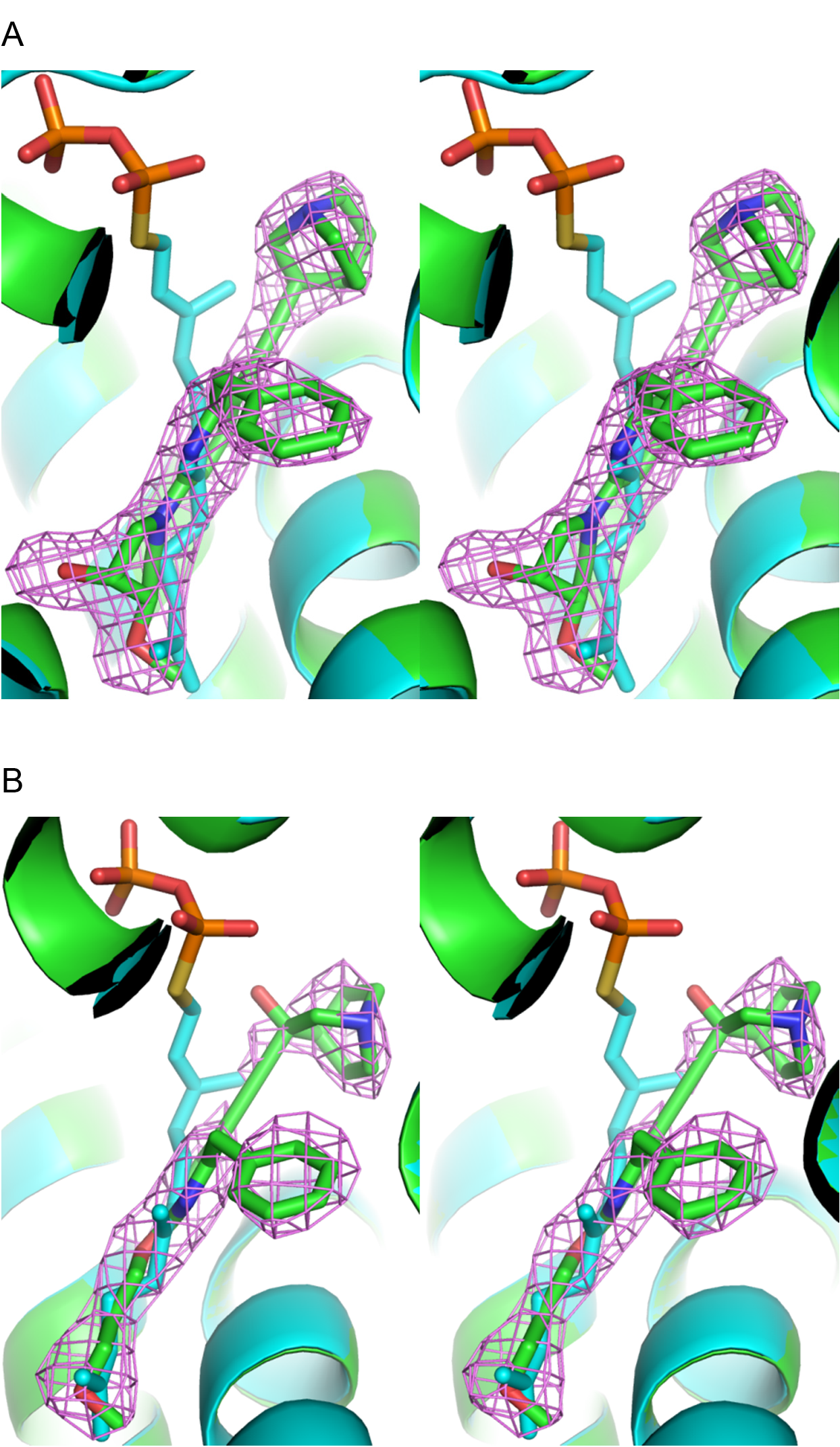

Supplement: Figure S7 — Stereo-view of electron densities of quinuclidines bound to TcSQS. (A) E5700 and (B) ER119884. The Fo-Fc “OMIT” maps are contoured at 3.0 σ level. The corresponding models are superimposed and shown as cartoon for the protein and sticks for the ligand, with the carbon atoms colored in green. For comparison, the aligned structure of TcSQS/FsPP is also shown, as cartoon and sticks with cyan carbons. (TIF) [file ppat.1004114.s007.tif]

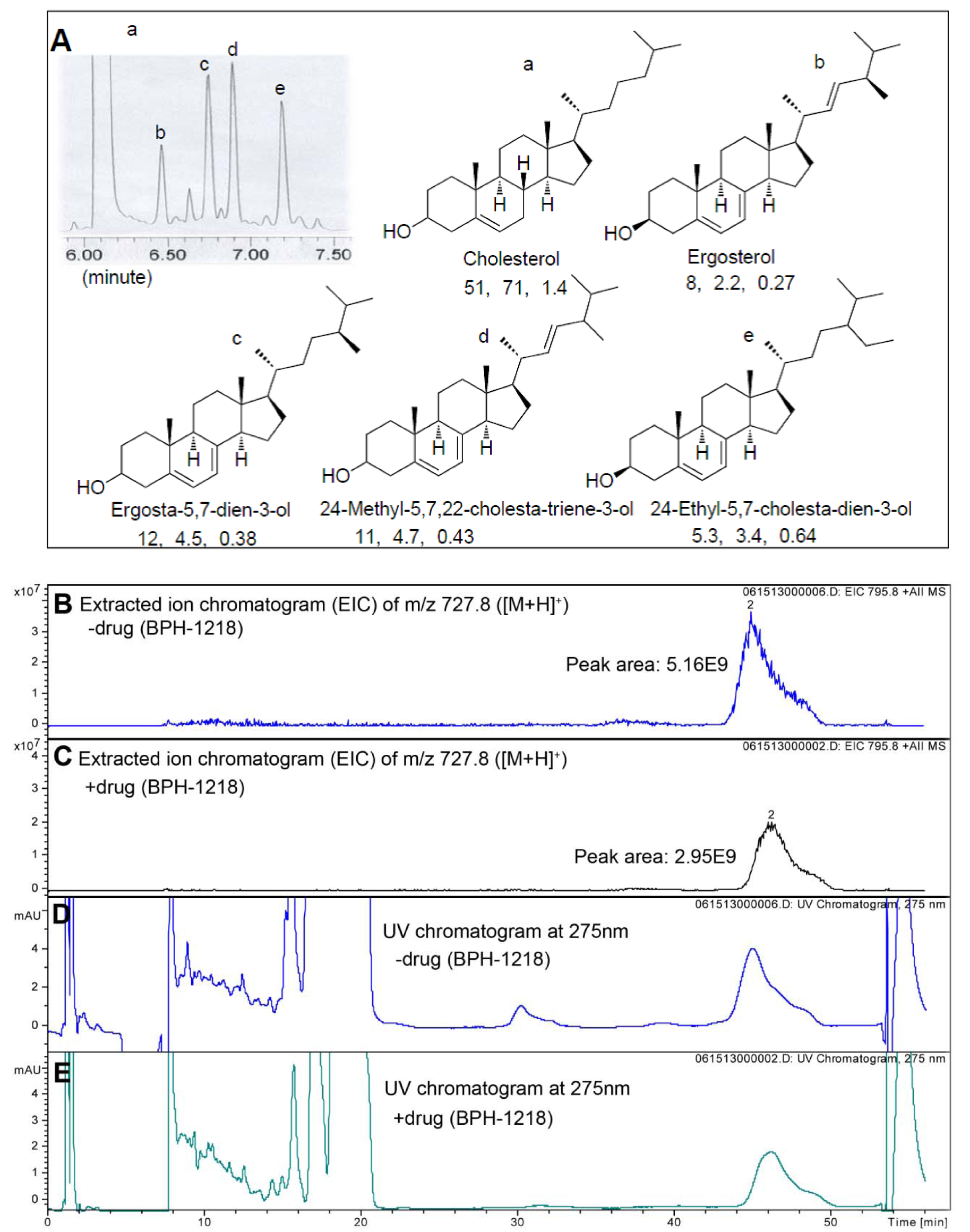

Supplement: Figure S8 — Effects of BPH-1218 on ergosterol and ubiquinone biosynthesis in T. cruzi epimastigotes. (A) GC-MS trace for trimethylsilyated sterols and sterol compositions (+drug %; −drug % and +drug/−drug ratios). (B)–(E) LC-MS and LC-UV results for UQ9 biosynthesis inhibition. (B, C). LC-MS without (B) or with (C) BPH-1218 in the growth medium. (D, E) LC-UV without (D) or with (E) BPH-1218 in the growth medium. (TIF) [file ppat.1004114.s008.tif]
